# Transmission dynamics and forecasts of the COVID-19 pandemic in Mexico, March-December 2020

**DOI:** 10.1371/journal.pone.0254826

**Published:** 2021-07-21

**Authors:** Amna Tariq, Juan M. Banda, Pavel Skums, Sushma Dahal, Carlos Castillo-Garsow, Baltazar Espinoza, Noel G. Brizuela, Roberto A. Saenz, Alexander Kirpich, Ruiyan Luo, Anuj Srivastava, Humberto Gutierrez, Nestor Garcia Chan, Ana I. Bento, Maria-Eugenia Jimenez-Corona, Gerardo Chowell

**Affiliations:** 1 Department of Population Health Sciences, School of Public Health, Georgia State University, Atlanta, GA, United States of America; 2 Department of Computer Science, College of Arts and Sciences, Georgia State University, Atlanta, GA, United States of America; 3 Department of Mathematics, Eastern Washington University, Cheney, Washington, United States of America; 4 Biocomplexity Institute and Initiative, Network Systems Science and Advanced Computing Division, University of Virginia, Charlottesville, Virginia, United States of America; 5 Scripps Institution of Oceanography, University of California San Diego, La Jolla, CA, United States of America; 6 Facultad de Ciencias, Universidad de Colima, Colima, Mexico; 7 Department of Statistics, Florida State University, Tallahassee, Florida, United States of America; 8 Department of Physics, Centro Universitario de Ciencias Exactas e Ingenierias (CUCEI), University of Guadalajara, Guadalajara, Mexico; 9 Department of Epidemiology and Biostatistics, School of Public Health, Indiana University Bloomington, Indiana, United States of America; 10 Department of Epidemiology, National Institute of Cardiology "Ignacio Chavez", Mexico City, Mexico; BronxCare Health System, Affiliated with Icahn School of Medicine at Mount Sinai, NY, USA, UNITED STATES

## Abstract

Mexico has experienced one of the highest COVID-19 mortality rates in the world. A delayed implementation of social distancing interventions in late March 2020 and a phased reopening of the country in June 2020 has facilitated sustained disease transmission in the region. In this study we systematically generate and compare 30-day ahead forecasts using previously validated growth models based on mortality trends from the Institute for Health Metrics and Evaluation for Mexico and Mexico City in near real-time. Moreover, we estimate reproduction numbers for SARS-CoV-2 based on the methods that rely on genomic data as well as case incidence data. Subsequently, functional data analysis techniques are utilized to analyze the shapes of COVID-19 growth rate curves at the state level to characterize the spatiotemporal transmission patterns of SARS-CoV-2. The early estimates of the reproduction number for Mexico were estimated between *R*_*t*_ ~1.1–1.3 from the genomic and case incidence data. Moreover, the mean estimate of *R*_*t*_ has fluctuated around ~1.0 from late July till end of September 2020. The spatial analysis characterizes the state-level dynamics of COVID-19 into four groups with distinct epidemic trajectories based on epidemic growth rates. Our results show that the sequential mortality forecasts from the GLM and Richards model predict a downward trend in the number of deaths for all thirteen forecast periods for Mexico and Mexico City. However, the sub-epidemic and IHME models perform better predicting a more realistic stable trajectory of COVID-19 mortality trends for the last three forecast periods (09/21-10/21, 09/28-10/27, 09/28-10/27) for Mexico and Mexico City. Our findings indicate that phenomenological models are useful tools for short-term epidemic forecasting albeit forecasts need to be interpreted with caution given the dynamic implementation and lifting of social distancing measures.

## Introduction

The ongoing COVID-19 (coronavirus disease 2019) pandemic is the most important global health challenge since the 1918 influenza pandemic that was caused by an A/H1N1 virus of avian origin [1, 2]. The severity of the COVID-19 pandemic calls for scientists, health professionals, and policymakers to collaboratively address the challenges posed by this lethal infectious disease. The causative SARS-CoV-2 (severe acute respiratory syndrome virus 2) is a novel, unusually complex, and highly transmissible virus that spreads via respiratory droplets and aerosols [3, 4]. It presents a clinical spectrum that ranges from asymptomatic individuals to conditions that require the use of mechanical ventilation to multiorgan failure and septic shock leading to death [3]. The ongoing COVID-19 pandemic has not only exerted significant morbidity but also an excruciating mortality burden with more than 79.2 million cases and 1.7 million deaths reported worldwide as of December 29, 2020 [5]. Approximately 27 countries globally including 9 countries in the Americas have reported more than 10,000 deaths attributable to SARS-CoV-2 as of December 29, 2020, despite the implementation of social distancing policies to limit the death toll [6]. In comparison, a total of 774 deaths were reported during the 2003 SARS multi-country epidemic and 858 deaths were reported during the 2012 MERS epidemic in Saudi Arabia [7, 8].

Determining the best containment strategies for the COVID-19 pandemic is a highly active research area [4]. While multiple vaccines against the novel coronavirus have begun to roll out amidst emerging SARS-CoV-2 variants, many scientific uncertainties exist that will dictate how vaccination campaigns will affect the course of the pandemic. For instance, it is still unclear if the vaccine will prevent the transmission of SARS-CoV-2 and its variants or just protect against more severe disease outcomes and death [9–11]. In these circumstances, non-pharmaceutical interventions remain the most promising policy levers to reduce virus transmission [12]. The epidemiological and mathematical models can help quantify the effects of non-pharmaceutical interventions that require behavioral changes such as washing hands, wearing facemasks and social distancing mandates to contain the spread of the virus [13, 14]. However, recent studies have demonstrated that population indicators such as poverty, population density, over-crowding, and inappropriate workplace conditions hinder the social distancing interventions propagating the unmitigated spread of the virus, especially in developing countries [15, 16]. Moreover, the differential mortality trends are also influenced by the disparate disease burden driven by the socioeconomic gradients with the poorest areas showing the highest preventable mortality rates [17].

Mexico, exhibiting one of the highest COVID-19 mortality impacts in the world thus far [18], is a highly populated country [19] with ~42% of the population living in poverty (defined as the state of a person or group of people that lack a specified amount of money or material possessions) [20] and ~60% of the population work in the informal sector [21]. A previous study reported an all-cause excess mortality of 26.10 per 10,000 population from March 1, 2020 to January 2, 2021, reflecting a heavy mortality impact of the COVID-19 pandemic in Mexico [22]. In this context, Mexico ranks fourth in the world in terms of the number of COVID-19 deaths, a tally surpassed only by the USA, Brazil, and India [23]. Moreover, the overall lethality of COVID-19 in Mexico has been reported to be 9.2% [24] accompanied by one of the highest death tolls among healthcare workers (~2500 deaths) [25] and the lowest number of COVID-19 tests per capita as of December 29, 2020 [26].

As the virus infiltrated the country, Mexican Ministry of Health identified three phases of the contingency plan: viral importation, community transmission, and epidemic to combat the COVID-19 pandemic in Mexico [27]. The pandemic in Mexico was likely seeded by imported COVID-19 cases reported by the government on February 28, 2020 [14, 28]. As the virus spread across the nation in phase one of the pandemic, some universities switched to virtual classes and some festivals and sporting events were postponed [29]. However, the government initially downplayed the impact of the virus and did not enforce strict social distancing measures [30]. This led to large gatherings at some social events such as concerts, festivals, and soccer tournaments amidst sustained disease transmission in the country [31]. A study conducted in Mexico estimated the early reproduction number for the first ten days of the pandemic between 2.9–4.9 [32]. However, the true impact of the pandemic was generally underestimated by the Mexican government despite active virus transmission in the country [33].

As local clusters of the disease started to appear in the community, phase 2 (community transmission) of the pandemic was declared on March 24, 2020 [34]. Authorities suspended all non-essential activities including the closure of public and entertainment places and banned gatherings of more than 100 people [34–36]. This was followed by the declaration of a national emergency on March 30, 2020. The new measures to fight the virus under the national emergency included extending the suspension of non-essential activities and a reduction in the number of people who can gather not to exceed fifty [37]. However, as the virus paved its way across the country ravaging the poor and rural communities, the government urged the public to comply with the stay-at-home orders [36, 38, 39]. These preventive orders from the government were met with mixed reactions from people belonging to different socio-economic sectors of the community [40]. Moreover, transportation restrictions to and from the regions most affected by COVID-19 were not implemented until April 16, 2020 [41]. Shortly after, on April 21, 2020, Mexico announced phase 3 of the contingency (epidemic phase) as widespread community transmission intensified [42].

With lockdowns and other restrictions in place, Mexican officials shared model output [43] predicting that COVID-19 case counts would peak in early May and that the pandemic was expected to end before July 2020 [44]. Despite notorious disagreement between surveillance data and government forecasts, these model predictions continued to be cited by official and independent sources [45, 46]. The extent to which these overly optimistic predictions skewed the plans and budgets of private and public institutions remains unknown. Under the official narrative that the pandemic would soon be over, Mexico planned a gradual phased re-opening of its economy in early June 2020, as the “new normal” phase [33, 47].

In Mexico, the reopening of the economic activities started on June 1 under a four-color traffic light monitoring system to alert the residents of the epidemiological risks of COVID-19 based on the level of severity of the pandemic in each state, on a bi-weekly basis [48]. As of December 29, 2020, Mexico exhibits high estimates of cumulative COVID-19 cases and deaths; 1,401,529 and 123,845 respectively [18]. Given the high transmission potential of the virus and limited application of tests in the country, testing only 24.54 people for every 1000 people (as of December 28, 2020) [26], estimates of the effective reproduction number from the case incidence data and near real-time epidemic projections using mortality data could prove to be highly beneficial to understand the trajectory of the COVID-19 pandemic in Mexico. It may also be useful to assess the effect of mobility patterns and intervention strategies such as the stay-at-home orders on the epidemic curve and understand the different spatiotemporal dynamics of the virus.

In order to investigate the transmission dynamics of the unfolding COVID-19 pandemic in Mexico, we analyze the case incidence data by date of symptoms onset and mortality data by date of report utilizing mathematical models that are useful to characterize the empirical patterns of epidemics [49, 50]. We estimate the effective reproduction number of SARS-CoV-2 in Mexico to understand the transmission dynamics of the virus and examine the mobility trends in relation to the curve of the number of COVID-19 deaths. Moreover, we employ statistical methods from functional data analysis to study the shapes of the COVID-19 growth rate curves at the state level. This helps us characterize the spatiotemporal dynamics of the pandemic based on the shape features of these curves. Lastly, Twitter data corresponding to frequency of tweets indicating stay-at-home-order are analyzed in relation to the COVID-19 case counts at the national level.

## Methods

### Data

Five sources of data are analyzed in this manuscript. A brief description of the datasets and their sources is presented below.

#### (i) IHME data for short-term forecasts

We utilized the openly published smoothed trend in daily COVID-19 reported deaths from the Institute of Health Metrics and Evaluation (IHME) for (i) Mexico (country) and (ii) Mexico City (capital of Mexico) as of October 9, 2020, to generate the sequential forecasts [51]. IHME smoothed death data estimates (current projection scenario) publicly available from the IHME COVID-19 estimates downloads page were analyzed [51]. The death estimates were corrected for the irregularities in the reporting of daily deaths by averaging model results over the last seven days. The statistical procedure of spline regressions obtained from MR-BRT (“meta-regression—Bayesian, regularized, trimmed”) was utilized to smooth the trend in COVID-19 reported deaths as described in the study [12]. This source of data for prediction modeling was chosen for its consistent updates. For this analysis, deaths reported by the IHME model (current projection scenario) on November 11, 2020, were used as a proxy for actual reported deaths attributed to COVID-19.

#### (ii) Apple mobility trends data

Publicly available mobility data for Mexico, published by Apple’s mobility trends reports was retrieved as of December 5, 2020 [52]. This aggregated and anonymized data is updated daily and includes the relative volume of directions requests per country compared to a baseline volume on January 13, 2020. Apple has released the data for the three modes of human mobility: driving, walking and public transit. The mobility measures are normalized in the range 0–100 for each country at the beginning of the series, so trends are relative to this baseline.

#### (iii) Case incidence and genomic data for estimating reproduction number

To estimate the reproduction number, we use two different data sources. For estimating the early reproduction number from the genomic data, 111 SARS-CoV-2 genome samples were obtained from the “global initiative on sharing avian influenza data” (GISAID) repository between February 27- May 29, 2020 [53]. For estimating the reproduction number from the case incidence data (early reproduction number and the instantaneous reproduction number), we utilized a publicly available time series of laboratory-confirmed cases by dates of symptoms onset which were obtained from the Mexican Ministry of Health Mexico, as of December 5, 2020 [18].

#### (iv) Case incidence data for spatial analysis

We recovered daily case incidence data for all 32 states of Mexico from March 20 to December 5 from the Ministry of Health Mexico, as of December 5, 2020 [18].

#### (v) Twitter data for Twitter analysis

For the Twitter data analysis, we retrieved data from the publicly available Twitter data set of COVID-19 chatter from March 12 to November 11, 2020 [54].

### Modeling framework for forecast generation

We harness three dynamic phenomenological growth models previously applied to multiple infectious diseases (e.g., SARS, foot and mouth disease, Ebola [55, 56] and the current COVID-19 outbreak [57, 58]) for mortality modeling and short-term forecasting in Mexico and Mexico City. These models include the simple scalar differential equation models such as the generalized logistic growth model [56] and the Richards growth model [59]. We also utilize the sub-epidemic wave model [55] which accommodates complex epidemic trajectories by assembling the contribution of multiple overlapping sub-epidemic waves. The mortality forecasts obtained from these mathematical models can provide valuable insights on the disease transmission mechanisms, the efficacy of intervention strategies and help evaluate optimal resource allocation procedures to inform public health policies. The COVID-19 mortality forecasts for Mexico and Mexico City generated by IHME (current projections scenario) are used as a benchmark model. The description of these models is provided in the S1 File.

Cumulative mortality forecasts obtained from our phenomenological growth models are compared with the total mean smoothed death data estimates retrieved from the IHME reference scenario and two IHME counterfactual scenarios. The IHME reference scenario depicts the “current projection”, which assumes that the social distancing measures are re-imposed for six weeks whenever daily deaths reach eight per million. The second scenario “mandates easing” implies what would happen if the government continued to ease social distancing measures without re-imposition. Lastly, the third scenario, “universal masks” accounts for universal facemask wearing, which reflects 95% facemask usage in public and social distancing mandates reimposed at 8 deaths per million. A detailed description of these modeling scenarios and their assumptions is explained in reference [12]. Moreover, the total mean smoothed death data estimates reported by the IHME reference scenario as of November 11, 2020, are considered as a proxy for the actual death count for each forecasting period.

### Model calibration and forecasting approach

We conducted 30-day ahead short-term forecasts utilizing thirteen data sets spanned over a period of four months (July 4-October 9, 2020) (Table 1). Each forecast was fitted to the daily death counts from the IHME smoothed death data estimates reported between March 20-September 27, 2020 for (i) Mexico and (ii) Mexico City. The first model calibration process relies on fifteen weeks of data, from March 20-July 4, 2020. Sequentially models are recalibrated each week with the most up-to-date data, meaning the length of the calibration period increases by one week up to August 2, 2020. However, owing to the irregular publishing of data estimates by the IHME, the length of the calibration period increased by 2 weeks after August 2, 2020. This was followed by a one-week increase from August 17-September 27, 2020, as the data estimates were again published every week.

**Table 1:** Characteristics of the data sets used for the sequential calibration and forecasting of the COVID-19 pandemic in Mexico and Mexico City (2020).

Date of the retrieval of the data set (MMDD)	Calibration period for the GLM, sub-epidemic, Richards and IHME model	Calibration period (number of days)	Forecast period for the GLM, sub-epidemic, Richards and IHME model
07/04	03/20-07/04	107	07/05-08/03
07/10	03/20-07/11	114	07/12-08/10
07/17	03/20-07/17	120	07/18-08/16
07/27	03/20-07/25	128	07/26-08/24
08/06	03/20-08/02	136	08/03-09/01
08/22	03/20-08/17	151	08/18-09/16
08/27	03/20-08/22	156	08/23-09/21
09/02	03/20-08/30	164	08/31-09/30
09/11	03/20-09/07	172	09/08-10/08
09/18	03/20-09/13	179	09/14-10/13
09/24	03/20-09/20	185	09/21-10/21
10/02	03/20-09/27	193	09/28-10/27
10/09	03/20-09/27	193	09/28-10/27

The 30-day ahead forecasts generated by calibrating our three phenomenological growth models with the IHME smoothed death data estimates are compared with the forecasts generated by the IHME reference scenario for the same calibration and forecasting periods.

For each of the three models; GLM, Richards growth model, and the sub-epidemic wave model, we estimate the best fit solution for each model using nonlinear least-square fitting procedure [60]. This process yields the best set of parameter estimates $\hat{\Theta }=({\hat{\theta }}_{1},{\hat{\theta }}_{2},\ldots ,{\hat{\theta }}_{m})$ by minimizing the sum of squared errors between the model fit, $f(t,\hat{\Theta })$ and the smoothed death data estimates, $y_{t_{i}}$. The estimated set of parameters $\hat{\Theta }=argmin\sum_{t=1}^{n}(f(t,\hat{\Theta }{{)-y}_{t_{i}})}^{2}$ define the best-fit model $f(t,\hat{\Theta })$. Here $\hat{\Theta }=(r,p,k_{o},q\;and\;C_{thr})$ corresponds to the set of parameters of the sub-epidemic model, $\hat{\Theta }=(r,a,k_{0})$ corresponds to the set of parameters of the Richards model, and $\hat{\Theta }=(r,p,k_{0})$ corresponds to the set of parameters of the GLM model [61]. For the GLM and sub-epidemic wave model, we provide initial best guesses of the parameter estimates. However, for the Richards growth model, we initialize the parameters for the nonlinear least-squares’ method [60] over a wide range of plausible parameters from a uniform distribution using Latin hypercube sampling [62]. This allows us to test the uniqueness of the best fit model. Moreover, the initial conditions are set at the first data point for each of the three models [61]. Uncertainty bounds around the best-fit solution are generated using a parametric bootstrap approach which involves resampling with replacement of data assuming a Poisson error structure for the GLM and sub-epidemic model. A negative binomial error structure is used to generate the uncertainty bounds for the Richards growth model; where we assume the mean to be three times the variance based on the noise in the data. A detailed description of this method is provided in the previous study [61].

Each of the *M* best-fit parameter sets is used to construct the 95% confidence intervals for each parameter by refitting the models to each of the *M* = 300 datasets generated by the bootstrap approach during the calibration phase. Further, each *M* best-fit model solution is used to generate *m* = 30 additional simulations with Poisson error structure for GLM and sub-epidemic model and negative binomial error structure for Richards model extended through a 30-day forecasting period. For the forecasting period, we construct the 95% prediction intervals with these 9000 (*M × m*) curves. A detailed description of the methods of parameter estimation can be found in prior studies [61, 63, 64].

### Performance metrics

We utilized the following four performance metrics to assess the quality of our model fit and the 30-day ahead short-term forecasts: the mean absolute error (MAE) [65], the mean squared error (MSE) [66], the coverage of the 95% prediction intervals (95% PI) [66], and the mean interval score (MIS) [66] for each of the three models. For calibration performance, we compare the model fit to the observed smoothed death data estimates fitted to the model, whereas for the performance of forecasts, we compare our forecasts with the smoothed death data estimates (current projections scenario) reported on November 11, 2020, for the time-period of the forecast.

The MSE and the MAE assess the average deviations of the model fit to the observed death data. The MAE is given by:

$$MAE=\frac{1}{n}\sum_{i=1}^{n}\left|f(t_{i},\hat{\Theta })-y_{t_{i}}\right|$$

The MSE is given by:

$$MSE=\frac{1}{n}\sum_{i=1}^{n}(f(t_{i},\hat{\Theta })-y_{t_{i}}{)}^{2}$$

where $y_{t_{i}}$ is the time series of reported smoothed death estimates, *t*_*i*_ is the time stamp and $\hat{\Theta }$ is the set of model parameters. For the calibration period, *n* equals the number of data points used for calibration, and for the forecasting period, *n =* 30 for the 30-day ahead short-term forecast.

Moreover, to assess the model uncertainty and performance of the prediction interval coverage, we use the 95% PI and MIS. The prediction interval coverage is defined as the proportion of observations that fall within 95% PI and is calculated as:

$$PI\;coverage=\frac{1}{n}\sum_{t=1}^{n}I\{y_{t_{i}}>L_{t_{i}}\cap y_{t_{i}}<U_{t_{i}}\}$$

where $y_{t_{i}}$ are the smoothed death data estimates, $L_{t_{i}}$ and $U_{t_{i}}$ are the lower and upper bounds of the 95% prediction intervals, respectively, *n* is the length of the period, and I is an indicator variable that equals 1 if the value of $y_{t_{i}}$ is in the specified interval and 0 otherwise.

The MIS addresses the width of the prediction interval as well as the coverage. The MIS is given by:

$$MIS=\frac{1}{n}\sum_{i=1}^{n}\left(U_{t_{i}}-L_{t_{i}}\right)+\frac{2}{0.05}(L_{t_{i}}-y_{t_{i}})I\{y_{t_{i}}<L_{t_{i}}\}+\frac{2}{0.05}(y_{t_{i}}-U_{t_{i}})I\{y_{t_{i}}>U_{t_{i}}\}$$

In this equation $L_{t_{i}},\;U_{t_{i}},\;y_{t_{i}}$, *n* and I are as specified above for PI coverage. Therefore, if the PI coverage is 1, the MIS is the average width of the interval across each time point. For two models that have an equivalent PI coverage, a lower value of MIS indicates narrower intervals [66].

### Mobility data analysis

In order to analyze the time-series data for Mexico from March 20-December 5, 2020 for three modes of mobility; driving, walking, and public transport, we utilize the R code developed by Healy [67]. We analyze the mobility trends to look for any common pattern with the mortality curve of COVID-19. The time series for mobility requests is decomposed into trends, weekly and remainder components. The trend is a locally weighted regression fitted to the data and the remainder is any residual leftover on any given day after the underlying trend and normal daily fluctuations have been accounted for.

### Reproduction number

We estimate the reproduction number, *R*_*t*_, for the early ascending phase of the COVID-19 pandemic in Mexico and the instantaneous reproduction number *R*_*t*_ throughout the pandemic. Reproduction number, *R*_*t*_, is a key parameter that characterizes the average number of secondary cases generated by a primary case at calendar time *t* during the outbreak. This quantity is critical to identify the intensity and magnitude of public health interventions required to contain a pandemic [68–70]. Estimates of *R*_*t*_ indicate if widespread disease transmission continues (*R*_*t*_>1) or disease transmission declines (*R*_*t*_<1). Therefore, to contain an outbreak, it is vital to maintain *R*_*t*_<1.

### Estimating the reproduction number, *R*_*t*_, from case incidence using generalized growth model (GGM)

We estimate the reproduction number by calibrating the GGM (as described in the S1 File) to the early growth phase of the pandemic (February 27-May 29, 2020) [71]. The generation interval of SARS-CoV-2 is modeled assuming gamma distribution with a mean of 5.2 days and a standard deviation of 1.72 days [72]. We estimate the growth rate parameter *r*, and the deceleration of growth parameter, *p*, as described in the S1 File. The GGM model is used to simulate the progression of local incidence cases *I*_*i*_ at calendar time *t*_*i*_. This is followed by the application of the discretized probability distribution of the generation interval, denoted by *ρ*_*i*_, to the renewal equation to estimate the reproduction number at the time *t*_*i*_ [73–75]:

$$R_{t_{i}}=\frac{I_{i}}{\sum_{j=0}^{i}{(I}_{i-j}\rho_{j})}$$

The numerator represents the total new cases *I*_*i*_ at time *t*_*i*_, and the denominator represents the total number of cases that contribute (as primary cases) to generate the new cases *I*_*i*_ (as secondary cases) at time *t*_*i*_. This way, *R*_*t*_, represents the average number of secondary cases generated by a single case at calendar time *t*. The uncertainty bounds around the curve of *R*_*t*_ are derived directly from the uncertainty associated with the parameter estimates (*r*, *p*) obtained from the GGM. We estimate *R*_*t*_ for 300 simulated curves assuming a negative binomial error structure [61].

### Instantaneous reproduction number *R*_*t*_, using the Cori method

The instantaneous reproduction number, *R*_*t*_, is estimated by the ratio of the number of new infections generated at calendar time *t* (*I*_*t*_), to the total infectiousness of infected individuals at time *t* given by $\sum_{s=1}^{t}I_{t-s}w_{s}$ [76, 77]. Hence *R*_*t*_ can be written as:

$$R_{t}=\frac{I_{t}}{\sum_{s=1}^{t}I_{t-s}w_{s}}$$

In this equation, *I*_*t*_ is the number of new infections on day *t* and *w*_*s*_ represents the infectivity function, which is the infectivity profile of the infected individual. This is dependent on the time since infection (*s)*, but is independent of the calendar time (*t*) [78, 79].

The term $\sum_{s=1}^{t}I_{t-s}w_{s}$ describes the sum of infection incidence up to time step *t* − 1, weighted by the infectivity function *w*_*s*_. The distribution of the generation time can be applied to approximate *w*_*s*_, however, since the time of infection is rarely an observed event, it is difficult to measure the distribution of generation time [76]. Therefore, the time of symptom onset is usually used to estimate the distribution of serial interval (SI), which is defined as the time interval between the dates of symptom onset among two successive cases in a disease transmission chain [80].

The infectiousness of a case is a function of the time since infection, which is proportional to *w*_*s*_ if the timing of infection in the primary case is set as time zero of *w*_*s*_ and we assume that the generation interval equals the SI. The SI was assumed to follow a gamma distribution with a mean of 5.2 days and a standard deviation of 1.72 days [72]. Analytical estimates of *R*_*t*_ were obtained within a Bayesian framework using EpiEstim R package in R language [80]. *R*_*t*_ was estimated at weekly intervals. We reported the median and 95% credible interval (CrI).

### Estimating the reproduction number, *R*, from the genomic analysis

In order to estimate the reproduction number for SARS-CoV-2 between February 27- May 29, 2020 from the genomic data, 111 SARS-CoV-2 genomes sampled from infected patients from Mexico, and their sampling times were obtained from GISAID repository [53]. Short sequences and sequences with a significant number of gaps and non-identified nucleotides were removed, yielding 83 high-quality sequences. For clustering, they were complemented by sequences from other geographical regions, down sampled to n = 4325 representative sequences. We used the sequence subsample from Nextstrain (www.nextstrain.org) global analysis as of August 15, 2020. These sequences were aligned to the reference genome taken from the literature [81] using MUSCLE [82] and trimmed to the same length of 29772 bp. The maximum likelihood phylogeny has been constructed using RAxML (Randomize Axelerated Maximum Likelihood) [83].

The largest Mexican cluster that possibly corresponds to within-country transmissions has been identified using hierarchical clustering of sequences. The phylodynamics analysis of that cluster has been carried out using BEAST v1.10.4 (Bayesian Evolutionary Analysis by sampling trees) [84]. We used a strict molecular clock and the tree prior with exponential growth coalescent. Markov Chain Monte Carlo sampling has been run for 10,000,000 iterations, and the parameters were sampled every 1000 iterations. The exponential growth rate *f* estimated by BEAST was used to calculate the reproductive number *R*. For that, we utilized the standard assumption that SARS-CoV-2 generation intervals (times between infection and onward transmission) are gamma-distributed [85]. In that case *R* can be estimated as $R={\left(1+\frac{f\sigma^{2}}{\mu }\right)}^{\frac{\mu^{2}}{\sigma^{2}}}$, where *μ* and *σ* are the mean and standard deviation of that gamma distribution. Their values were taken from the study [72].

### Spatial analysis

For the shape analysis of incidence rate curves, we followed reference [86] to pre-process the daily cumulative COVID-19 case data at the state level as follows:

- Time differencing: If *f*_*i*_(*t*) denotes the given cumulative number of confirmed cases for state *i* on day *t*, then per day growth rate at time *t* is given by *g*_*i*_(*t*) = *f*_*i*_(*t*)−*f*_*i*_(*t*−1).
- Smoothing: We then smooth the normalized curves using the smooth function in MATLAB.
- Rescaling: Rescaling of each curve is done by dividing each *g*_*i*_ by the total confirmed cases for a state *i*. That is, compute *h*_*i*_(*t*) = *g*_*i*_(*t*)/*r*_*i*_, where *r*_*i*_ = ∑_*t*_*g*_*i*_(*t*).

This process is depicted in S17 Fig. To identify the clusters by comparing the curves, we used a simple metric. For any two rate curves, *h*_*i*_ and *h*_*j*_, we compute the norm ||*h*_*i*_−*h*_*j*_||, where the double bars denote the L^2^ norm of the difference function, i.e., ||*h*_*i*_−*h*_*j*_|| = $\sqrt{\sum_{t}{\left(h_{i}(t)-h_{j}(t)\right)}^{2}}$. To perform clustering of 32 curves into smaller groups, we apply the dendrogram function in Matlab using the “ward” linkage as explained in reference [86]. The number of clusters is decided empirically based on the display of overall clustering results. After clustering the states into different groups, we derived the average curve for each cluster after using a time wrapping algorithm as discussed in prior studies [86, 87].

### Twitter data analysis

To observe any relationship between the COVID-19 cases by date of symptoms onset and the frequency of tweets indicating stay-at-home orders we used a public dataset of 698 million tweets of COVID-19 chatter [54]. The frequency of tweets indicating stay-at-home order is used to gauge the compliance of people with the orders of staying at home to avoid the spread of the virus by maintaining social distance. Tweets indicate the magnitude of the people being pro-lockdown and depict how these numbers have dwindled over the course of the pandemic. To get to the plotted data, we removed all retweets and tweets that were not in the Spanish language. We also filtered the tweets by the following hashtags: #quedateencasa, and #trabajardesdecasa, which are two of the most used hashtags when users refer to the COVID-19 pandemic and their engagement with health measures. Lastly, we limited the tweets to the ones that originated from Mexico, via its 2-letter country code: MX. A set of 521,359 unique tweets were gathered from March 12 to November 11, 2020. We then overlay the curve of tweets over the epidemic curve in Mexico to observe any relationship between the shape of the epidemic trajectory and the shape of the curve for the frequency of tweets during the established time period. We also estimate the correlation coefficient between the cases and frequency of tweets.

## Results

**Fig 1:**
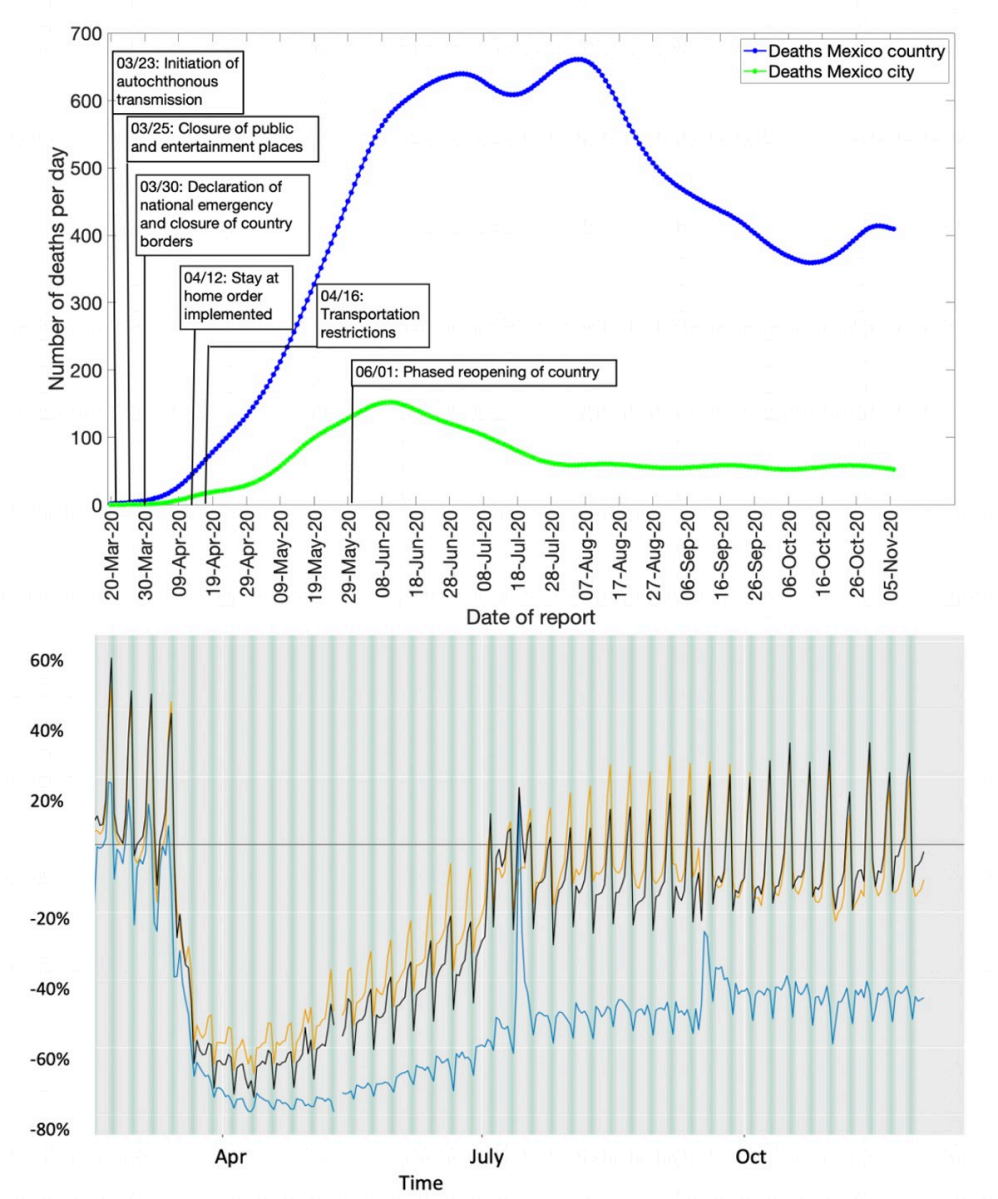
Upper panel: Epidemic curve for the COVID-19 deaths in Mexico and Mexico City from March 20-November 11, 2020. The blue line depicts the confirmed deaths in Mexico and the green line depicts the confirmed deaths in Mexico City. Lower panel: The mobility trends for Mexico from February 28-December 5, 2020. The orange line shows the driving trend, the blue line shows the transit trend, and the black line shows the walking trend.

As of November 11, 2020, Mexico has reported 105,656 deaths whereas Mexico City has reported 15,742 deaths per IHME smoothed death data estimates. Fig 1 (upper panel) shows the daily COVID-19 death curve in Mexico and Mexico City from March 20-November 11, 2020. The mobility trend for Mexico (Fig 1, lower panel) shows that the human mobility tracked in the form of walking, driving and public transportation declined from the end of March to the beginning of June, corresponding to the implementation of social distancing interventions and the *Jornada Nacional de Sana Distancia* that was put in place between March 23-May 30, 2020 enforcing the suspension of non-essential activities in public, private and social sectors [88]. The driving and walking trends subsequently increased in June with the reopening of the non-essential services. Fig 1 (upper panel) shows that the reopening of the country coincides with the highest levels of daily deaths. These remain at a high level for just over two months (June and July). Then from mid-August, the number of deaths begins to fall, reaching a reduction of nearly 50% by mid-October. However, at the end of October 2020, a new spurt in death counts can be observed.

In the subsequent sections, we first present the results for the short-term forecasting, followed by the estimation of the reproduction numbers. Then we present the results for spatial analysis and Twitter data analysis.

### Model calibration and forecasting performance

Here we compare the calibration and 30-day ahead forecasting performance between March 20- September 27, 2020, and July 5-October 27, 2020 respectively of the three models: the GLM, Richards growth model, and the sub-epidemic wave model for (i) Mexico and (ii) Mexico City. We also compare the results of our cumulative mortality forecasts with the total mean smoothed death data estimates retrieved from the three IHME model scenarios (as explained in the methods section).

### Calibration performance

**Fig 2:**
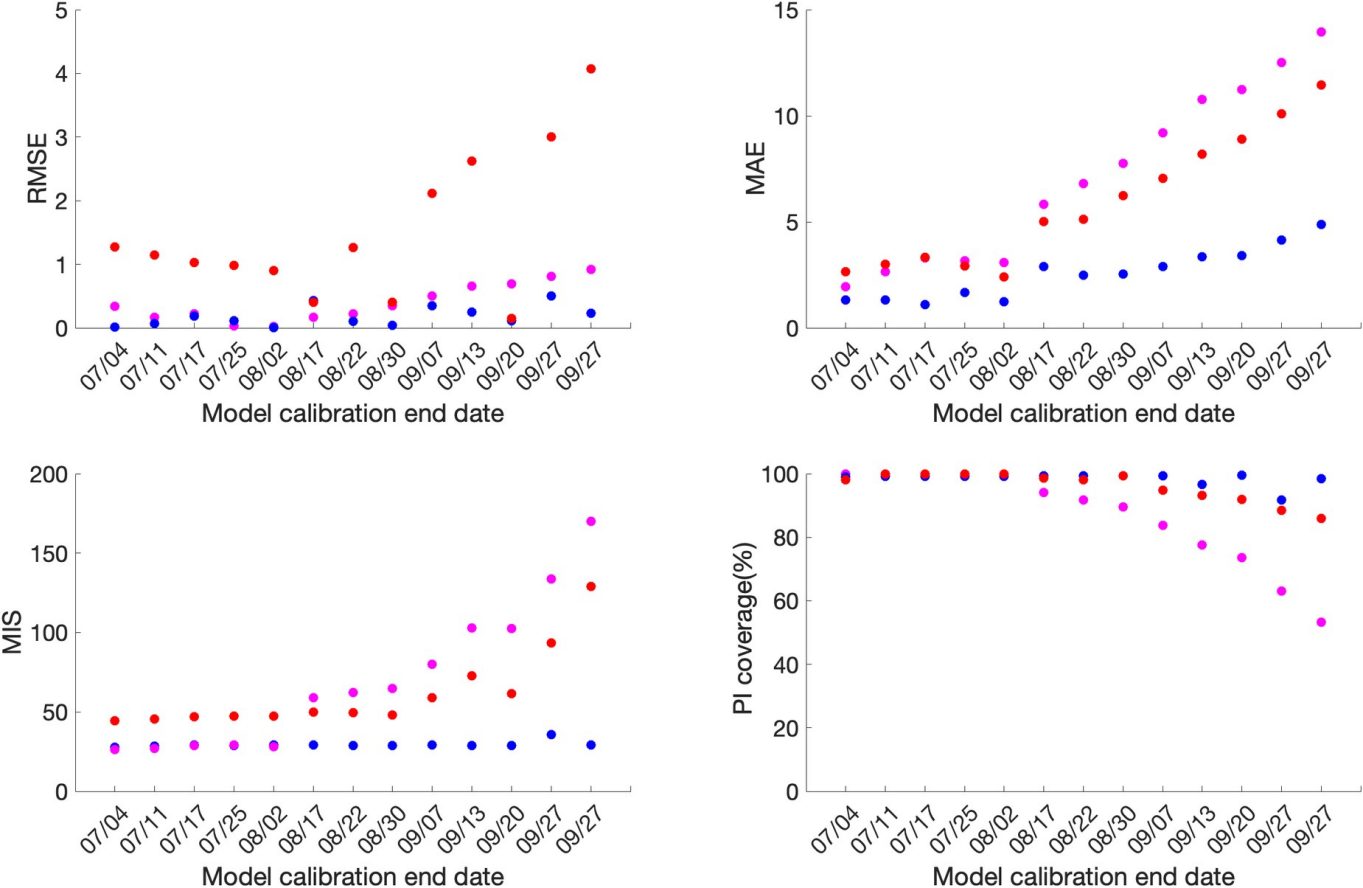
**Calibration performance for each of the thirteen sequential calibration phases for GLM (magenta), Richards (red), and sub-epidemic (blue) model for Mexico.** High 95% PI coverage and lower mean interval score (MIS), root mean square error (RMSE), and mean absolute error (MAE) indicate better performance.

Across the thirteen sequential model calibration phases for Mexico over a period of seven months (March-September), as provided in S1 Table in S1 File and Fig 2, the sub-epidemic model outperforms the GLM with lower RMSE estimates for the seven calibration phases 03/20-07/04, 03/20-07/17, 03/20-08/17, 03/20-08/22, 03/20-09/13, 03/20-09/20, 03/20-09/27. The GLM model outperforms the other two models for the remaining six calibration phases in terms of RMSE. The Richards model has substantially higher RMSE (between 10.2–24.9) across all thirteen calibration phases indicating a sub-optimal model fit. The sub-epidemic model also outperforms the other two models in terms of MAE, MIS, and the 95% PI coverage. It has the lowest values for MIS and the highest 95% PI coverage for nine of the thirteen calibration phases (S1 Table in S1 File). Moreover, the sub-epidemic model has the lowest MAE for eleven calibration phases. The Richards model shows much higher MIS and lower 95% PI coverage compared to the GLM and sub-epidemic model, pointing towards a sub-optimal model fit.

**Fig 3:**
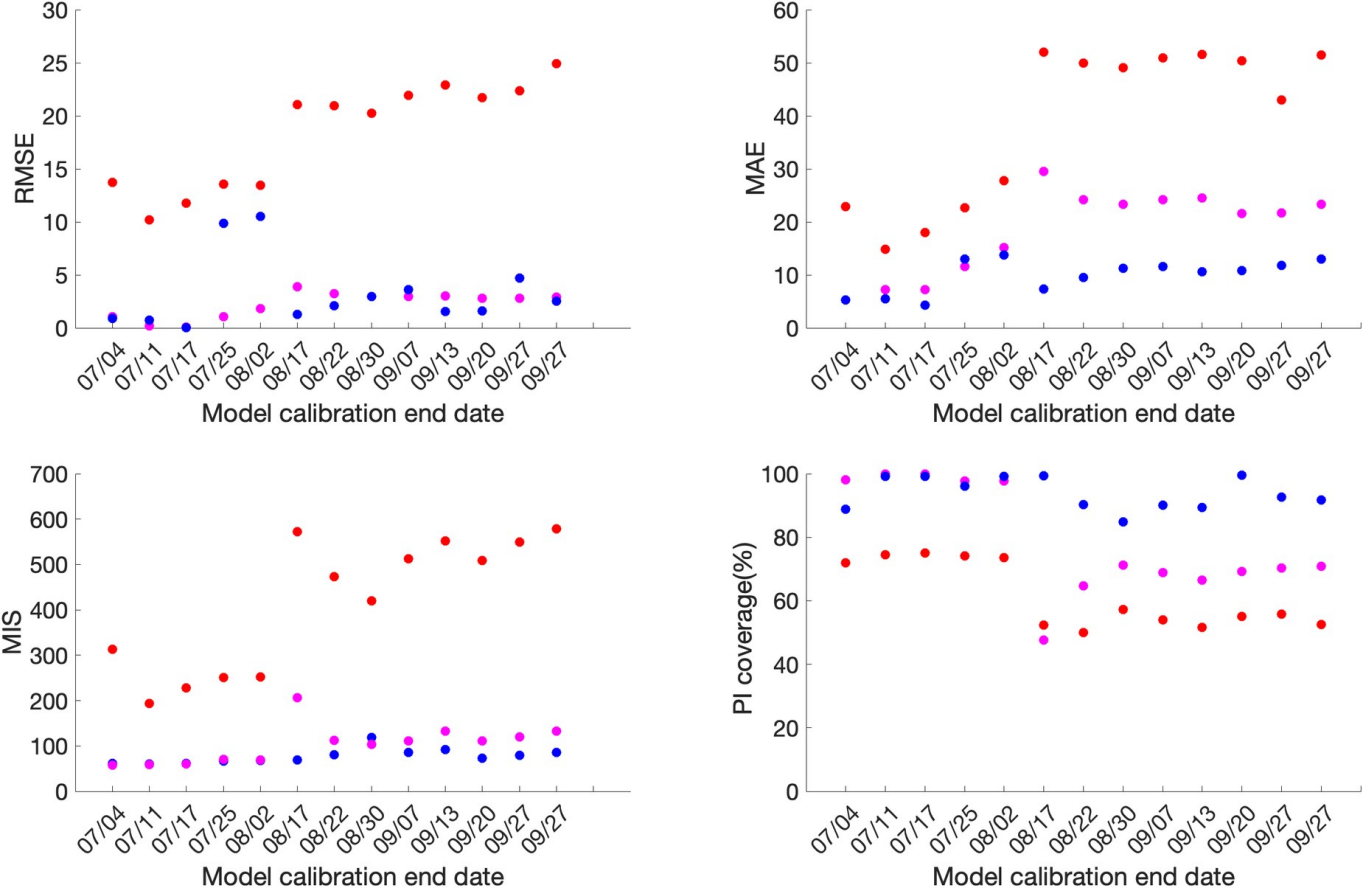
**Calibration performance for each of the thirteen sequential calibration phases for GLM (magenta), Richards (red), and sub-epidemic (blue) model for Mexico City.** High 95% PI coverage and lower mean interval score (MIS), root mean square error (RMSE) and mean absolute error (MAE) indicate better performance.

For Mexico City, the sub-epidemic model outperforms the other two models in terms of all performance metrics. It has the lowest RMSE for eleven of the thirteen calibration phases followed by the GLM and Richards model. The MAE is also the lowest for the sub-epidemic model for all thirteen calibration phases followed by the GLM and Richards growth model. Further, in terms of MIS, the sub-epidemic model outperforms the Richards and GLM model for nine calibration phases whereas the GLM model outperforms the other two models in the remaining four calibration phases (03/20-07/04, 03/20-07/11, 03/20-07/17, 03/20-08/02). The Richards model has much higher estimates for the MIS compared to the other two models indicating a sub-optimal model fit. The 95% PI coverage across all thirteen calibration phases lies between 91.6–99.4% for the sub-epidemic model, followed by the Richards model (85.9–100%) and the GLM model (53.2–100%) (S2 Table in S1 File, Fig 3).

Overall, the goodness of fit metrics points toward the sub-epidemic model as the most appropriate model for the Mexico City and Mexico across all four-performance metrics except for the RMSE for Mexico, where the estimates of the GLM model compete with the sub-epidemic model.

### Forecasting performance

**Fig 4:**
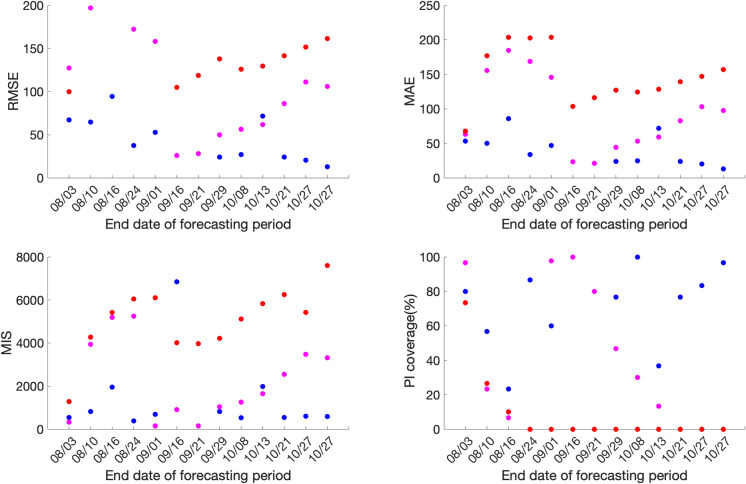
**Forecasting period performance metrics for each of the thirteen sequential forecasting phases for GLM (magenta), Richards (red) and sub-epidemic (blue) model for Mexico.** High 95% PI coverage and lower mean interval score (MIS), root mean square error (RMSE) and mean absolute error (MAE) indicate better performance.

For Mexico, the sub-epidemic model consistently outperforms the GLM and Richards growth model for ten out of the thirteen forecasting phases in terms of RMSE and MAE, eight forecasting phases in terms of MIS and nine forecasting phases in terms of the 95% PI coverage. This is followed by the GLM and the Richards growth model (Fig 4, S4 Table in S1 File).

**Fig 5:**
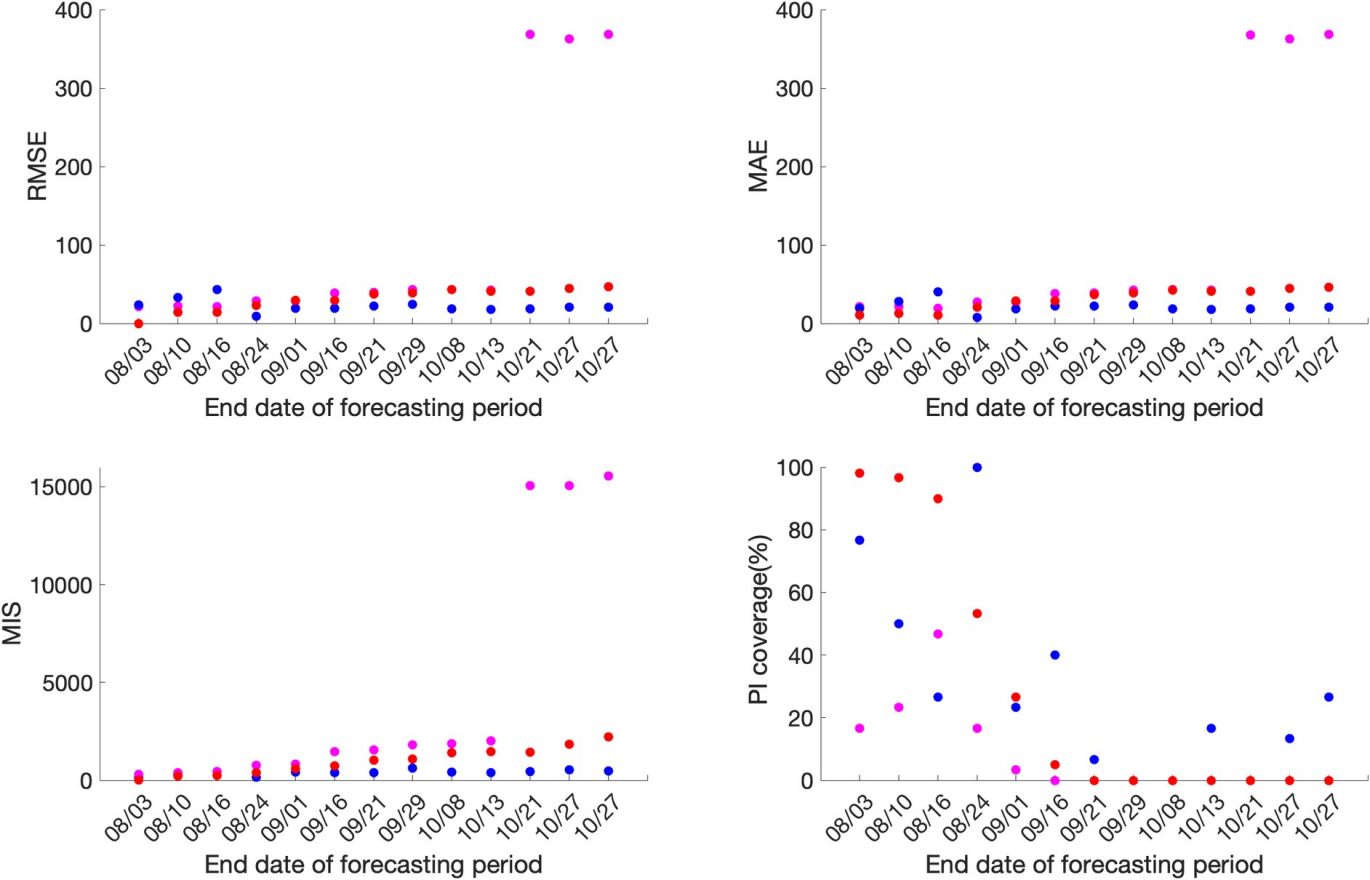
**Forecasting period performance metrics for each of the thirteen sequential forecasting phases for GLM (magenta), Richards (red) and sub-epidemic (blue) model for the Mexico City.** High 95% PI coverage and lower mean interval score (MIS), root mean square error (RMSE) and mean absolute error (MAE) indicate better performance.

Similarly, for Mexico City, the sub-epidemic model consistently outperforms the GLM and Richards growth model for ten of the thirteen forecasting phases in terms of RMSE and MAE and eleven forecasting phases in terms of the MIS. Whereas, in terms of 95% PI coverage, forecasting phases 08/31–09/29, 09/08-10/08 and 09/21-10/21 show zero 95% PI coverage across all three models. The sub-epidemic model outperforms the Richards and GLM model in six forecasting phases, with the Richards model performing better than the GLM model for the remaining four forecasting phases in terms of the 95% PI coverage (Fig 5, S3 Table in S1 File).

### Comparison of daily death forecasts

The thirteen sequentially generated daily death forecasts from GLM and Richards growth model, for Mexico and Mexico City indicate towards a sustained decline in the number of deaths (S1–S4 Figs). However, the IHME model forecasts (retrieved from smoothed death data estimates, current projections scenario) indicate a decline in the number of deaths for the first six forecast periods followed by a stable epidemic trajectory for the last seven forecasts, for Mexico City and Mexico. Unlike the GLM and Richards models, the sub-epidemic model can reproduce the observed stabilization of daily deaths observed after the first six forecast periods for Mexico and the last three forecast periods for Mexico City, as can also be seen with the IHME model (S5–S8 Figs).

### Comparison of cumulative mortality forecasts

**Fig 6:**
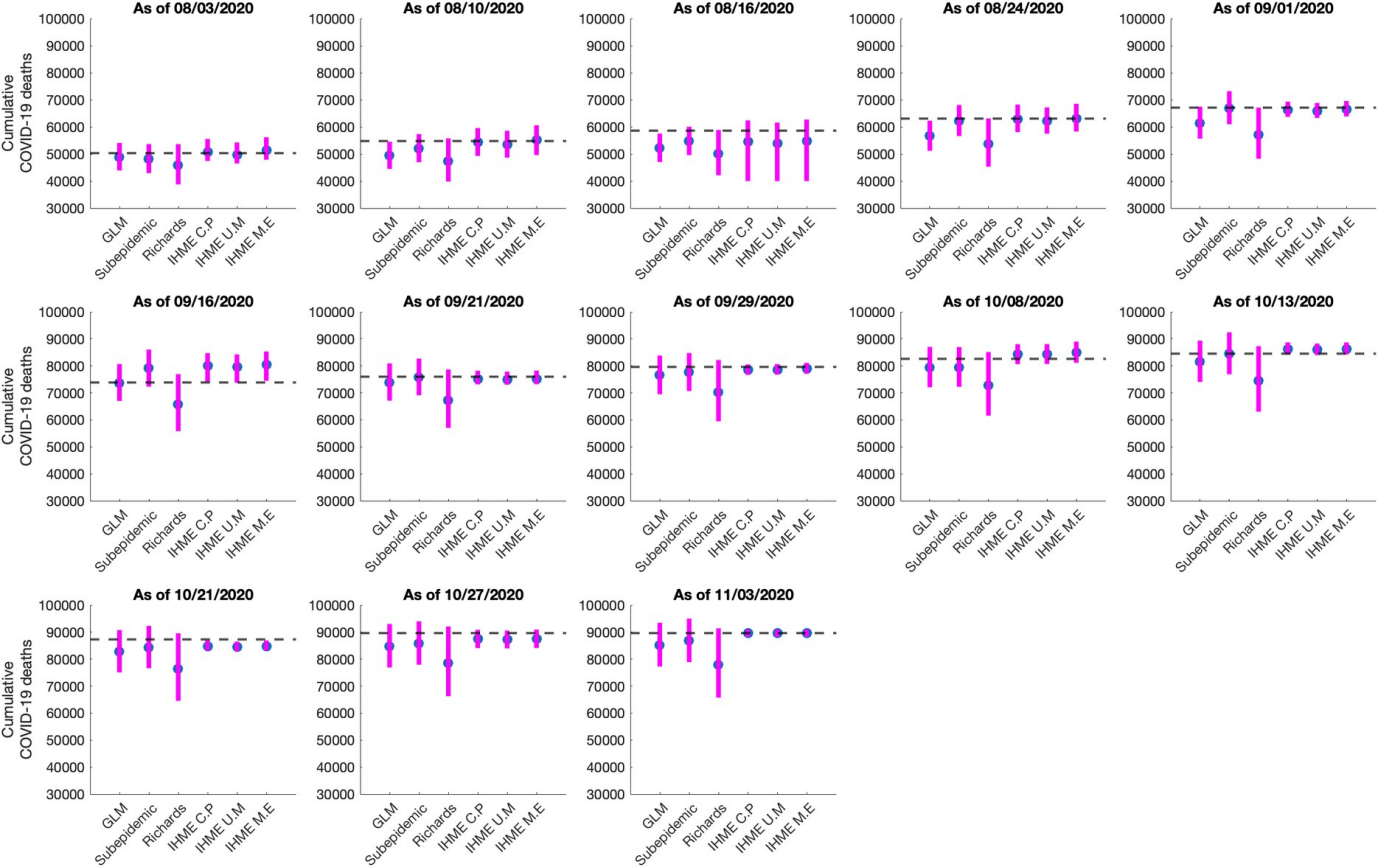
Systematic comparison of six models (GLM, Richards, sub-epidemic model, IHME current projections (IHME C.P), IHME universal masks (IHME U.M) and IHME mandates easing (IHME M.E) to predict the cumulative COVID-19 deaths for Mexico in the thirteen sequential forecasts. The blue circles represent the mean deaths, and the magenta vertical line indicates the 95% PI around the mean death count. The horizontal dashed line represents the actual death count reported by that date as published in the November 11, 2020, IHME estimates file.

**Fig 7:**
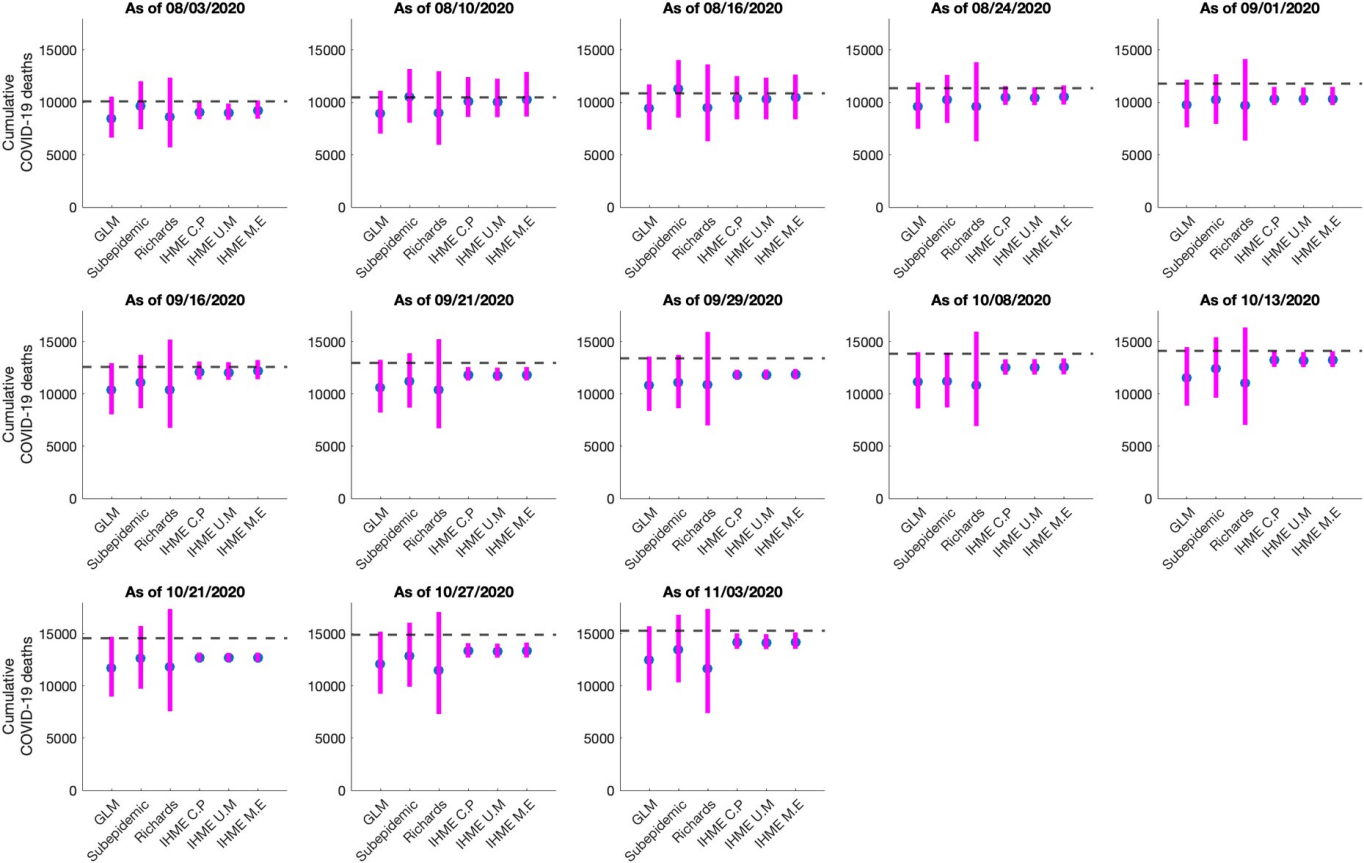
Systematic comparison of six models (GLM, Richards, sub-epidemic model, IHME current projections (IHME C.P), IHME universal masks (IHME U.M) and IHME mandates easing (IHME M.E) to predict the cumulative COVID-19 deaths for the Mexico City in the thirteen sequential forecasts. The blue circles represent the mean deaths, and the magenta vertical line indicates the 95% PI around the mean death count. The horizontal dashed line represents the actual death count reported by that date as published in the November 11, 2020, IHME estimates file.

The total number of COVID-19 deaths is an important quantity to measure the progression of an epidemic. Here we present the results of the estimated cumulative death counts obtained from our 30-day ahead cumulative forecasts generated using the GLM, Richards and sub-epidemic growth model. We compare these results with the total mean smoothed death data estimates obtained from the three IHME modeling scenarios; current projection, universal masks and mandates easing. The total mean smoothed death data estimates obtained from the IHME current projections scenario as of November 11, 2020, are considered as a proxy for the actual death count for each date that the cumulative forecast is obtained (Figs 6 and 7).

#### Mexico

The 30-day ahead cumulative forecast results for the thirteen sequentially generated forecasts for Mexico utilizing GLM, Richards growth model, sub-epidemic wave model, and the IHME model (current projections scenario) are presented in S9–S12 Figs. The cumulative mortality estimates comparison is given in Fig 6. For the first, second, third, and thirteenth generated forecast the GLM, sub-epidemic model, and the Richards model tend to underestimate the true deaths counts (~50,255, ~54,857, ~58,604, 89,730 deaths respectively), whereas the three IHME forecasting scenarios closely estimate the actual death counts for the first, second, and thirteenth forecasting periods. For the fourth, fifth, and seventh generated forecast the sub-epidemic model and the IHME scenarios most closely approximate the actual death counts (~63,078, ~67,075, ~76,054 deaths respectively). For the sixth generated forecast the GLM model closely approximates the actual death count (~73,911 deaths) whereas for the tenth generated forecast the sub-epidemic model closely approximates the actual deaths (~84,471 deaths). For the eighth, ninth, eleventh, and twelfth generated forecast, GLM, Richards, and sub-epidemic model tend to under-predict the actual death counts with the IHME model underestimating the actual death counts for the eleventh and twelfth generated forecast and overestimating the total death counts for the ninth generated forecast (Table 2).

**Table 2:** Cumulative mortality estimates obtained from the six models (GLM, Richards model, sub-epidemic model, IHME current projections, IHME universal mask and IHME mandates easing) at the end of each forecasting period for the COVID-19 pandemic in Mexico (2020).

Forecast Number	Forecast period (MMDD)	GLM Mean (95% PI)	Sub-epidemic model Mean (95% PI)	Richards model Mean (95% PI)	IHME current projections Mean (95% PI)	IHME universal mask Mean (95% PI)	IHME mandates easing Mean (95% PI)	Actual deaths reported as of Nov 11, 2020
1	07/05-08/03	48,917 (43,931–54,039	48,110 (42,939–53,661)	45,808 (38,808–53,665)	50,721 (47,410–55,597)	49,692 (46,500–54,250)	51,299 (47,893–56,184)	50,255
2	07/12-08/10	49,412 (44,517–49,412)	52,085 (46,973–57,379)	47,358 (39,836–55,808)	54,438 (49,269–59,598)	53,615 (48,634–58,590)	55,176 (49,609–60,621)	54,857
3	07/18-08/16	52,197 (47,059–57,541)	54,758 (49,600–60,070)	50,055 (42,161–58,892)	54,572 (39,989–62,409)	54,020 (39,989–61,614)	54,749 (39,989–62,710)	58,604
4	07/26-08/24	56,658 (51,208–62,320)	62,271 (56,644–68,073)	53,742 (45,332–63,144)	62,902 (58,094–68,253)	62,194 (57,516–67,205)	63,116 (58,285–68,542)	63,078
5	08/03-09/01	61,451 (55,655–67,494)	67,010 (60,988–73,219)	57,186 (48,270–67,114)	66,376 (63,705–69,334)	65,944 (63,308–68,853)	66,582 (63,865–69,612)	67,075
6	08/18-09/16	73,700 (66,996–80,655)	79,144 (72,306–86,048)	65,814 (55,834–76,954)	80,072 (74,140–84,710)	79,598 (73,772–84,225)	80,537 (74,479–85,288)	73,911
7	08/23-09/21	73,901 (67,126–80,909)	75,809 (69,107–82,699)	67,273 (57,061–78,667)	75,125 (73,161–78,209)	74,887 (72,993–77,883)	75,160 (73,207–78,254)	76,054
8	08/31-09/30	76,535 (69,509–83,826)	77,629 (70,688–84,743)	70,218 (59,490–82,174)	78,525 (76,644–80,538)	78,653 (76,767–80,669)	79,016 (77,057–81,135)	79,683
9	09/08-10/08	79,406 (72,084–87,022)	79,491 (72,250–86,959)	72,712 (61,556–85,135)	84,215 (80,639–88,038)	84,307 (80,682–88,069)	84,937 (81,130–88,999)	82,669
10	09/14-10/13	81,546 (74,030–89,356)	84,561 (76,905–92,411)	74,504 (63,026–87,292)	86,249 (84,255–88,722)	85,926 (83,982–88,256)	86,249 (84,259–88,694)	84,471
11	09/21-10/21	82,815 (75,098, 90,804)	84,392 (76,640–92,327)	76,386 (64,579–89,556)	84,731 (83,126–86,880)	84,435 (82,872–86,512)	84,731 (83,135–86,864)	87,396
12	09/28-10/27	84,827 (76,896–93,047)	85,885 (77,943–94,022)	78,448 (66,244–92,090)	87,491 (84,095–90,872)	87,265 (83,967–90,580	87,522 (84,115–90,945)	89,730
13	09/28-10/27	85,197 (77,258–93,454)	86,850 (78,896–95,001)	77,876 (65,750–91,401)	89,666 (88,264–91,036)	89,627 (88,280–91036)	89,667 (88,281–91,036)	89,730

In summary, the Richards growth model consistently under-estimates the actual death counts compared to the GLM, sub-epidemic model, and three IHME modeling scenarios. The GLM model also provides lower estimates of mean death counts compared to the sub-epidemic model and the three IHME modeling scenarios, but higher mean death estimates compared to the Richards model. The 95% PI for the Richards model is substantially wider than the other five models indicating greater uncertainty in the results. The actual mean death counts lie within the 95% PI of the sub-epidemic model for all the thirteen forecasts. Moreover, the three IHME modeling scenarios predict approximately similar cumulative death counts across the thirteen generated forecasts, indicating that the three scenarios do not differ substantially.

#### Mexico City

The 30 day ahead cumulative forecast results for thirteen sequentially generated forecasts for Mexico City utilizing GLM, Richards model, sub-epidemic wave model, and IHME model (current projections scenario) are presented in S13–S16 Figs. The cumulative death comparison is given in Fig 7 and Table 3. For the first generated forecast, the sub-epidemic model closely approximates the actual death count (~10,081 deaths). For the second generated forecast, the sub-epidemic model and the IHME scenarios closely approximate the actual death count (~10,496 deaths). For the third and sixth generated forecast, GLM and Richards model underestimate the actual death count (~10,859, ~12,615 deaths respectively) whereas the sub-epidemic model closely estimates the actual death count for the third forecast and under-predicts the actual death count for the sixth forecast. The three IHME model scenarios seem to predict the actual death counts closely. For the fourth, fifth, and seventh to thirteenth generated forecasts all models under-predict the actual death counts.

**Table 3:** Cumulative mortality estimates obtained from the six models (GLM, Richards model, sub-epidemic model, IHME current projections, IHME universal mask, and IHME mandates easing) at the end of each forecasting period for the COVID-19 pandemic in Mexico City (2020).

Forecast Number	Forecast period (MMDD)	GLM Mean (95% PI)	Sub-epidemic model Mean (95% PI)	Richards model Mean (95% PI)	IHME current projections Mean (95% PI)	IHME universal mask Mean (95% PI)	IHME mandates easing Mean (95% PI)	Actual deaths reported as of Nov 11, 2020
1	07/05-08/03	8,480 (6,642–10,549)	9,655 (7,437–12,016)	8,628 (5,712–12,363)	9,075 (8,334–9,888)	8,991 (8,334–9,888)	9,195 (8,443–10,182)	10,081
2	07/12-08/10	8,968 (7,022–11,119)	10,534 (8,063–13,187)	9,015 (5,951–12,971)	10,091 (8,607–12,421)	10,018 (8,598–12,263)	10,254 (8,648–12,905)	10,496
3	07/18-08/16	9,447 (7,402–11,710)	11,287 (8,541–14,037)	9,495 (6,291–13,616)	10,388 (8,382–12,505)	10,323 (8,381–12,365)	10,467 (8,381–12,660)	10,859
4	07/26-08/24	9,588 (7,478–11,891)	10,249 (8,042–12,622)	9,575 (6,283–13,836)	10,481 (9,761–11,551)	10,424 (9,729–11,433)	10,526 (9,791–11,623)	11,326
5	08/03-09/01	9,786 (7,621–12,166)	10,232 (7,950–12,686)	9,737 (6,351–14,140)	10,314 (9,746–11,477)	10,290 (9,733–11,423)	10,314 (9,746–11,477)	11,769
6	08/18-09/16	10,388 (8,054–12,957)	11,103 (8,646–13,752)	10,425 (6,762–15,212)	12,099 (11,387–13,118)	12,055 (11,362–13,046)	12,184 (11,422–13,255)	12,615
7	08/23-09/21	10,615 (8,226–13,272)	11,205 (8,700–13,911)	10,411 (6,719–15,250)	11,826 (11,289–12,584)	11,794 (11,273–12,527)	11,826 (11,290–12,585)	12,966
8	08/31-09/30	10,851 (8,381–13,581)	11,103 (8,646–13,752)	10,872 (6,997–15,950)	11,829 (11,397–12,328)	11,842 (11,409–12,527)	11,871 (11,421–12,394)	13,414
9	09/08-10/08	11,182 (8,621–14,011)	11,237 (8,721–13,955)	10,820 (6,936–15,966)	12,547 (11,851–13,318)	12,560 (11,859–13,340)	12,604 (11,881–13,413)	13,838
10	09/14-10/13	11,553 (8,887–14,492)	12,443 (9,645–15,439)	11,064 (7,043–16,373)	13,256 (12,586–14,106)	13,215 (12,566–14,031)	13,256 (12,857–14,105)	14,107
11	09/21-10/21	11,711 (8,985–14,714)	12,636 (9,737–15,742)	11,811 (7,578–17,367)	12,727 (12,326–13,200)	12,699 (12,310, 13,156)	12,728 (12,327–13,192)	14,561
12	09/28-10/27	12,074 (9,253–15,195)	12,878 (9,919–16,054)	11,503 (7,315–17,079)	13,358 (12,718–14,095)	13,332 (12,705–14,049)	13,361 (12,720–14,153)	14,911
13	09/28-10/27	12,493 (9,570–15,716)	13,460 (10,341–16,815)	11,659 (7,398–17,370)	14,172 (13,539–15,031)	14,131 (13,522–14,958)	14,191 (14,541–15,128)	15,306

In general, the Richards growth model has a much wider 95% PI coverage compared to the other models indicating greater uncertainty in the results. The mean cumulative death count estimates for the GLM and Richards model closely approximate each other. However, the actual mean death counts lie within the 95% PI of the GLM and sub-epidemic model for all thirteen forecasts. The three IHME model scenarios predict approximately similar cumulative death counts across the thirteen generated forecasts with much narrow 95% PI’s, indicating that the three scenarios do not differ substantially.

### Reproduction number

#### Estimate of reproduction number, *R*_*t*_ from case incidence data

**Fig 8:**
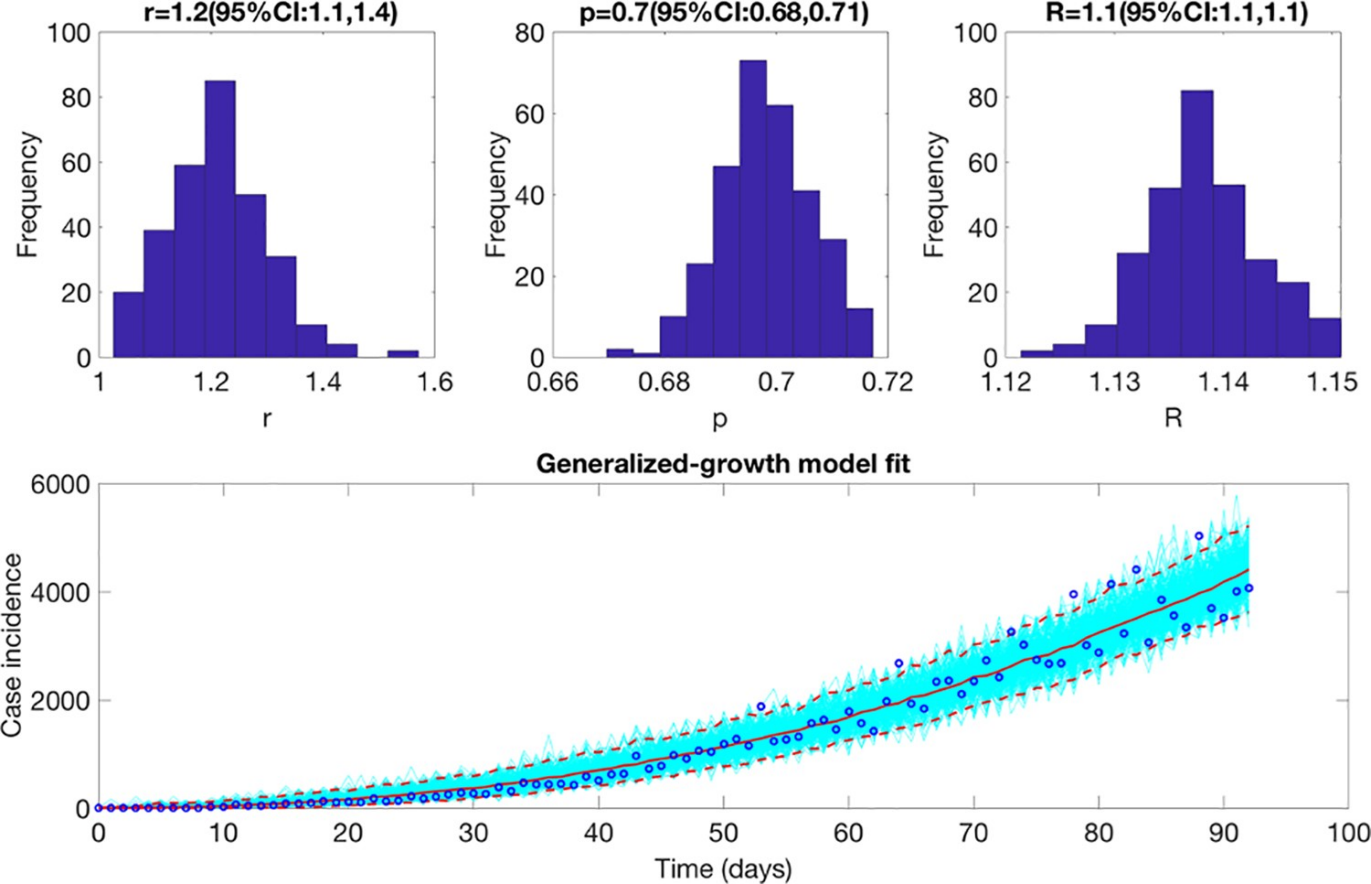
Upper panel: Reproduction number with 95% CI estimated using the GGM model. The estimated reproduction number of the COVID-19 pandemic in Mexico as of May 29, 2020, is 1.1 (95% CI: [1.1, 1.1]). The growth rate parameter, *r*, is estimated at 1.2 (95% CI: [1.1, 1.4]) and the deceleration of growth parameter, *p*, is estimated at 0.7 (95% CI: [0.68, 0.71]). Lower panel: The lower panel shows the GGM fit to the case incidence data for the first 90 days.

The reproduction number from the case incidence data (February 27- May 29, 2020) using GGM was estimated at ***R***_***t***_~1.1(95% CI: [1.1,1.1]). The growth rate parameter, *r*, was estimated at 1.2 (95% CI: [1.1, 1.4]) and the deceleration of growth parameter, *p*, was estimated at 0.7 (95% CI: [0.68,0.71]) indicating early sub-exponential growth dynamics of the pandemic (Fig 8).

#### Estimate of instantaneous reproduction number, R_t_

**Fig 9:**
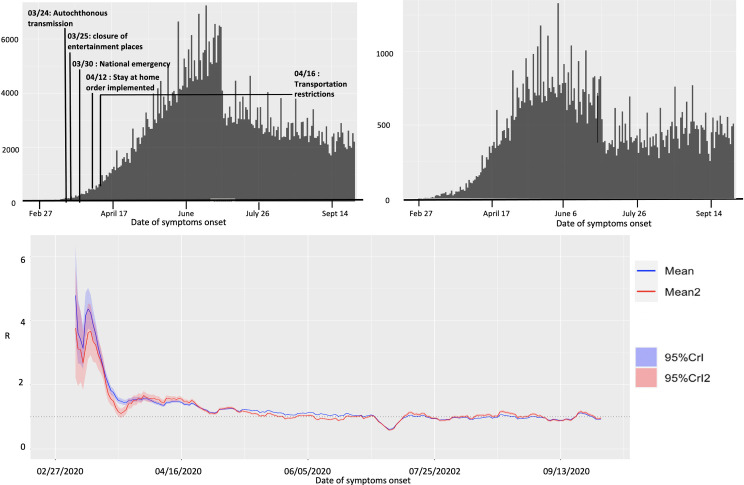
Upper panel: Epidemiological curve (by the dates of symptom onset) for Mexico (left panel) and Mexico City (right panel) as of September 27, 2020. Lower panel: Instantaneous reproduction number with 95% credible intervals for the COVID-19 pandemic in Mexico as of September 27, 2020. The red solid line represents the mean reproduction number for Mexico and the red shaded area represents the 95% credible interval around it. The blue solid line represents the mean reproduction number for Mexico City and the blue shaded region represents the 95% credible interval around it.

The instantaneous reproduction number for Mexico remained consistently above 1.0 until the end of May 2020, after which the reproduction number has fluctuated around 1.0 with the estimate of *R*_*t*_~0.93 (95% CrI: [0.91, 0.94]) as of September 27, 2020. For Mexico City, the reproduction number remained above 1.0 until the end of June after which it has fluctuated around 1.0 with the estimate of *R*_*t*_~0.96 (95% CrI: [0.93, 0.99]) as of September 27, 2020 (Fig 9).

#### Estimate of reproduction number, R from genomic data analysis

**Fig 10:**
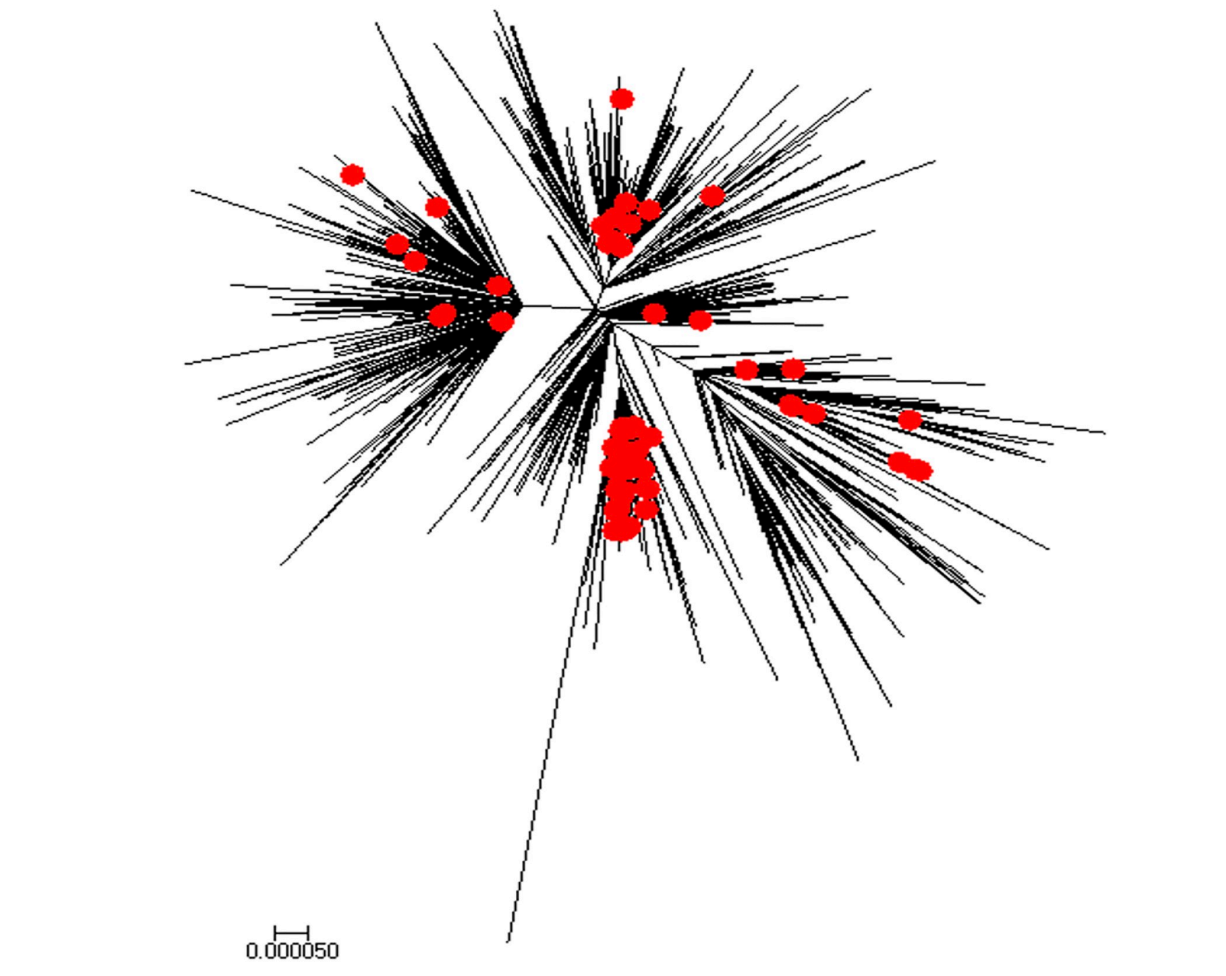
Global ML tree for SARS-CoV-2 genomic data from February 27- May 29, 2020. Sequences sampled in Mexico are highlighted in red.

The majority of analyzed Mexican SARS-CoV-2 sequences (69 out of 83) have been sampled in March and April 2020. These sequences are spread along the whole global SARS-CoV-2 phylogeny (Fig 10) and split into multiple clusters. This indicates multiple introductions of SARS-CoV-2 to the country during the initial pandemic stage (February 27- May 29, 2020). For the largest cluster of size 42, the reproduction number was estimated at *R* = 1.3 (95% HDP (Highest Posterior Density) interval [1.1,1.5]) in accordance with the early estimate of *R*_*t*_ obtained from the case incidence data.

### Spatial analysis

**Fig 11:**
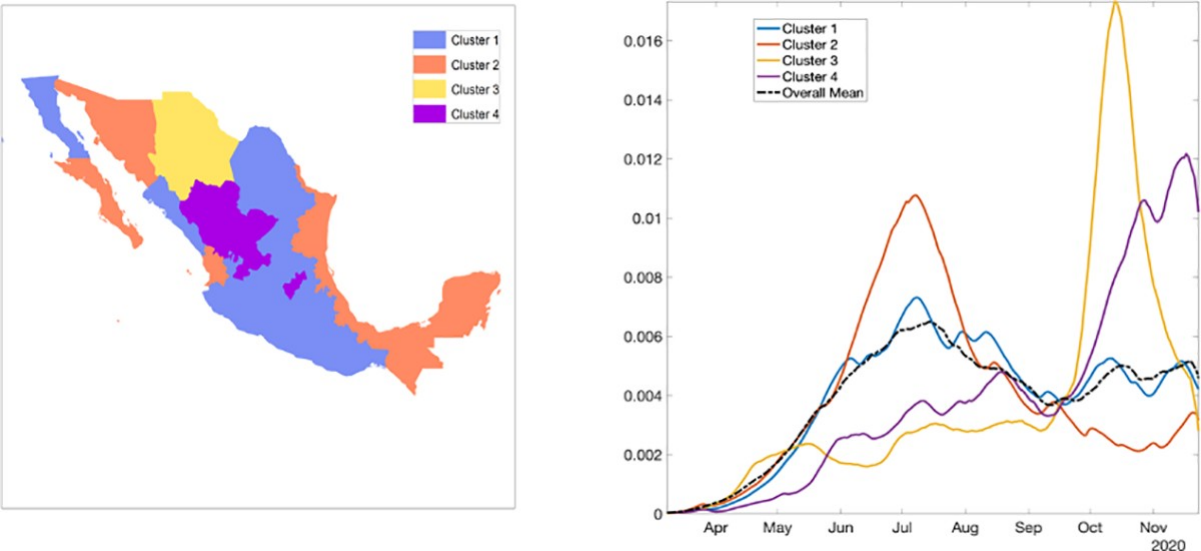
Clusters of states by their growth rates. Cluster 1 in blue, cluster 2 in orange, cluster 3 in yellow, and cluster 4 in purple. The right panel shows the average growth rate curves for each cluster (solid curves) and their overall average (black broken curve).

The results from pre-processing of COVID-19 data into growth rate functions are shown in S17 Fig. The dendrogram plot shown in S18 Fig presents the results of clustering and the states are color coded based on their cluster membership within the map of Mexico (Fig 11; left panel). The four predominant clusters that were identified include the following states:

Cluster 1: Baja California, Coahuila, Colima, Mexico City, Guanajuato, Guerrero, Hidalgo, Jalisco, Mexico, Michoacán, Morelos, Nuevo Leon, Oaxaca, Puebla, San Luis Potosi, Sinaloa, and Tlaxcala
Cluster 2: Baja California Sur, Campeche, Chiapas, Nayarit, Quintana Roo, Sonora, Tabasco, Tamaulipas, Veracruz, and Yucatan
Cluster 3: Chihuahua
Cluster 4: Aguascalientes, Durango, Queretaro, and Zacatecas

Fig 11 (right panel) shows the average shape of growth rate curves in each cluster and the overall cluster average. S19 Fig shows mean growth rate curves and one standard-deviation bands around it in each cluster. Since cluster 3 included only one state, the average growth rate curves of cluster 1, cluster 2, and cluster 4 are shown. The average growth patterns in the three categories are very distinct and clearly visible. For cluster 1, the rate rises rapidly from April to July and then shows small fluctuations. For cluster 2, there is a rapid increase in growth rate from April to July followed by a rapid decline. Chihuahua in cluster 3 shows a slow growth rate until September followed by a rapid rise until mid-September which then declines rapidly. For cluster 4, the rate rises slowly, from April to September, and then shows a rapid rise (S20 Fig).

**Fig 12:**
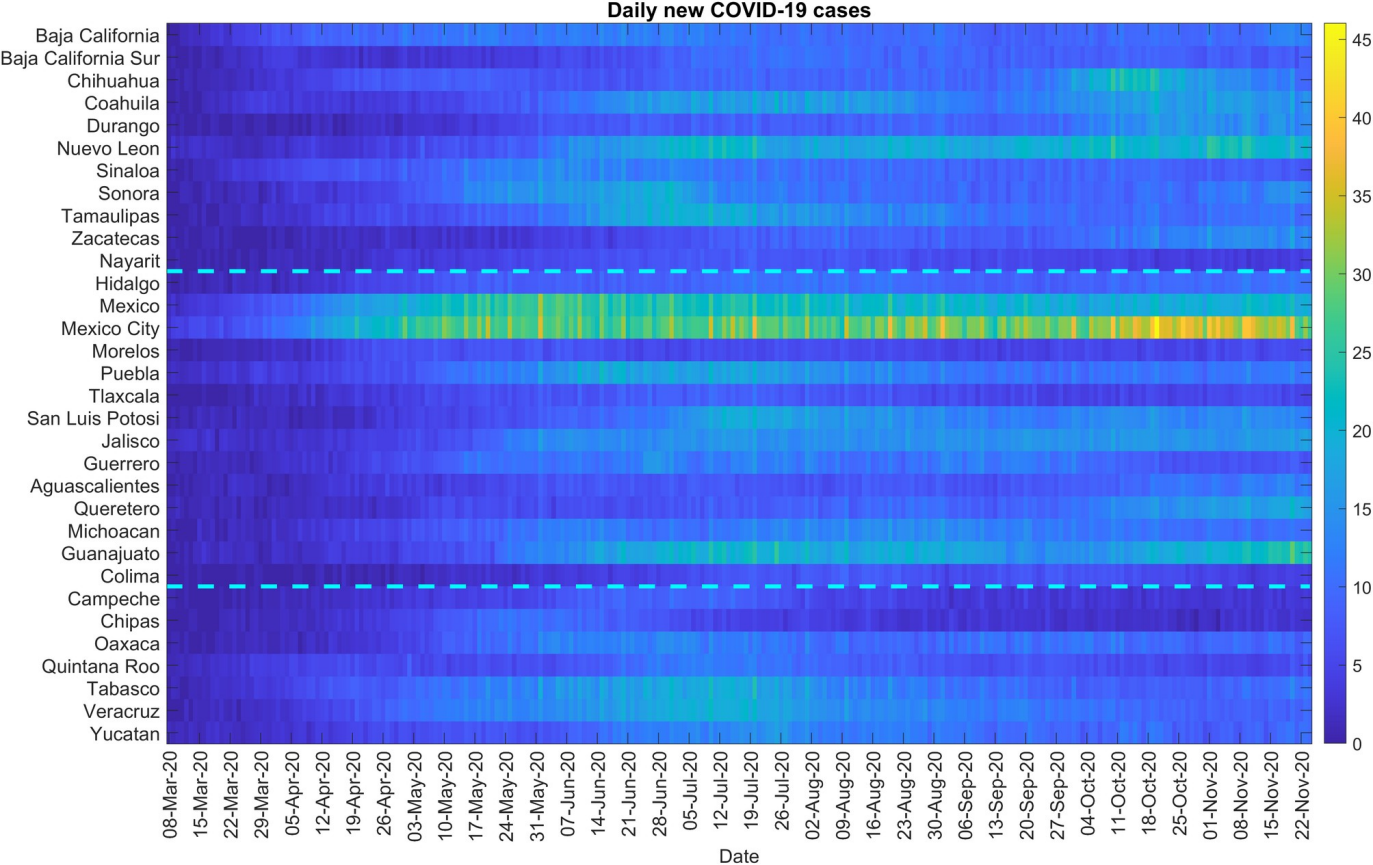
Color scale image of daily COVID-19 cases by region.

From the colormap (Fig 12) we can see that the cases were concentrated from the beginning in the central region in Mexico and Mexico City. Daily cases have been square root transformed to reduce variability in the amplitude of the time series while dashed lines separate the Northern, Central, and Southern regions. S20 Fig shows the time-series graph of daily COVID-19 new cases by the date for all states, Northern states, Central states, and the Southern states. As observed for both Northern and Central regions including the national level, the epidemic peaked in mid-July followed by a decline at around mid-September, which then started rising again. Southern states exhibit a stable decline. S21 Fig shows the total number of COVID-19 cases at the state level as of December 5, 2020. Some of the areas with a higher concentration of COVID-19 cases are Mexico City, Mexico state, Guanajuato in the central region and, Nuevo Leon in the Northern region.

### Twitter data analysis

The epidemic curve for Mexico is overlaid with the curve of tweets indicating stay-at-home orders in Mexico as shown in S22 Fig. The engagement of people in Mexico with the #quedateencasa hashtag (stay-at-home order hashtag) has been gradually declining as the number of cases has continued to increase or remain at a steady pace, showing the frustration and apathy of the public on lock downs and restrictions. Mostly the non-government public health experts are calling for more lockdowns or continued social distancing measures (without being heard by the authorities). It could also imply that the population is not following the government’s stay-at-home orders and hence we continue to observe the cases. S22 Fig shows that the highest number of tweets were made during the earlier part of the pandemic, with the number of tweets declining as of mid-May 2020. In contrast, the number of cases by onset dates peaked around mid-June. The correlation coefficient between the epidemic curve of cases by dates of onset and the curve of tweets representing the stay-at-home orders was estimated at R = -0.001 from March 12- November 11, 2020.

## Discussion

We report initial sub-exponential growth dynamics of the COVID-19 pandemic in Mexico and Mexico City with the deceleration of growth parameter, *p*, estimated between 0.6–0.8 from the case incidence and mortality data. Yet, the early estimates of reproduction number, *R*_*t*_, demonstrate the sustained disease transmission in the country. As *R*_*t*_ fluctuates around 1.0 since the end of July 2020, variable epidemic growth patterns can be observed at the national and state level. As the virus transmission continues in Mexico, Twitter analysis implies the relaxation of lockdowns with inconsequential decline in the mobility patterns observed over the last few weeks as evident from Apple’s mobility trends. Moreover, the systematic comparison of our models across thirteen sequential forecasts suggests that the sub-epidemic model is the most appropriate model for mortality forecasting. The sub-epidemic model can reproduce the stabilization in the trajectory of mortality forecasts as predicted by the IHME model.

The sub-exponential growth pattern of the COVID-19 pandemic in Mexico can be attributed to a myriad of factors including non-homogenous mixing, spatial structure, population mobility, behavior changes, and control interventions [89]. Our results are consistent with the sub-exponential growth patterns of COVID-19 outbreaks observed in Mexico [90] and Chile [91]. Along with the observed sub-exponential growth dynamics of the COVID-19 pandemic in Mexico, the reproduction number estimated from the genomic sequence analysis and the case incidence data (*R*_*t*_~ 1.1–1.2) indicate a sustained transmission of SARS-CoV-2 in Mexico during the early transmission phase of the virus (February 27- May 29, 2020). Our estimates of *R*_*t*_ are similar to the estimates of reproduction numbers retrieved from other studies conducted in Mexico [92], Chile [91, 93], Peru [94], and Brazil [95]. The early estimate of *R*_*t*_ obtained from the Cori *et al*. method (instantaneous reproduction number) in our study also coincides with the early estimates of *R*_*t*_ obtained from the case incidence data and the genomic data (*R*_*t*_~1). The instantaneous reproduction number estimated from our study shows that *R*_*t*_ is slightly above 1 since the end of March 2020, without a significant increase. This is in accordance with the estimates of *R*_*t*_ obtained from another study conducted in Mexico [14].

In general, Mexico has observed a sustained SARS-CoV-2 transmission and an increasing or sustained case load despite the implementation of social distancing interventions including the stay-at-home orders that were eased around June 2020. As our Twitter data analysis also shows, the number of cases by onset dates was negatively correlated to the stay-at-home orders. A possible explanation indicates that people might have stopped following the government’s preventive orders to stay at home as a result of pandemic fatigue [96, 97]. Mexico has been one of the countries where the stay-at-home orders have been least respected. The average reduction in mobility in Mexico was reported to be 80% by mid-April that has declined to ~34% since August 2020. In comparison, Argentina and Peru have showed the largest mobility reductions ranging from 60–90% between March-September 2020 [98]. The preventive orders have affected the Mexican population disproportionately, with some proportion of the population exhibiting aggression towards quarantine and stay-at-home orders [40]. However, the public health professionals seem to be frustrated towards the relaxation of stay-at-home orders and reopening of the country, as the cases and deaths keep mounting. We can also appreciate the variable spatiotemporal dynamics of the COVID-19 pandemic in Mexico. Our classification of the epidemic patterns at the state level in Mexico shows a distinct variation of growth rates across states. For instance, cluster 1 including Baja California, Colima, and Mexico City has stable growth at a higher rate and cluster 4 including Aguascalientes, Durango, Queretaro, and Zacatecas shows a rising pattern in the growth rate (Fig 11). Hence, the place of residence of an individual can largely influence their vulnerability during an epidemic [99]. This information can be utilized by the states in guiding their decision regarding the implementation of public health measures. For example, states in clusters 1 and 4 may need strict public health measures to contain the pandemic.

Appropriate short-term forecasts can also help gauge the impact of interventions in near real-time. In this study, we compared the performance of our three models for short-term real-time forecasting the COVID-19 mortality estimates in Mexico and Mexico City. As in Figs 2–5, the sub-epidemic model can be declared the most appropriate model as it exhibits the most desirable performance metrics across most of the calibration and forecasting phases. This model has the capacity to accommodate more complex epidemic trajectories suggesting a longer epidemic wave and can better adjust to the early signs of changes in disease transmission, while other models (GLM and Richards) are less reactive. This model can also be utilized as a potential forecasting tool for other cities in Mexico and be compared with other prediction models. Further short-term forecasts (5,10 days) could be also be conducted with the sub-epidemic model using the consecutive calibration phases to reduce the error metrics [55].

Overall, the sequential forecasts based on the daily smoothed death estimates for Mexico from the two models (GLM and Richards growth model) suggest a decline in overall deaths (S1 and S2 Figs) consistent with the sustained decline in COVID-19 associated case fatalities since mid-August as reported officially by the government of Mexico [100]. However, this decline in COVID-19 deaths can be attributed to the inaccurate reporting of deaths in the surveillance system or downplay of fatalities by the government. For instance, the reported excess deaths as of September 26, 2020, are estimated to be 193,170 with 139,151 deaths attributable to COVID-19 [101]. While the official tally of COVID-19 deaths in Mexico is only exceeded by the USA and Brazil, it is roughly the same as that of India, a country whose population is ten times larger than Mexico [102]. As observed earlier, the easing of the social distancing interventions and lifting of lockdowns in Mexico in the month of June led to a surge of the COVID-19 associated deaths [103]. In June, the government of Mexico also inaccurately forecasted that a potential decline in the number of COVID-19 deaths would be observed by September 2020 [104]. Therefore, the forecasting trends need to be interpreted cautiously to inform policies. The IHME model also shows a decline in COVID-19 deaths in Mexico from mid-August-September, which have stabilized since then for the last six forecast periods (S5 Fig). The sub-epidemic model also indicates a stabilization of the deaths for the last seven forecast periods (S6 Fig) consistent with the results obtained from the IHME model.

Similarly, for Mexico City, the sequential forecasts obtained from the GLM and Richards model fitted to the daily death data estimates indicate a decline in the overall deaths (S3 and S4 Figs). The IHME and sub-epidemic models on the other hand indicate a stabilization in the trajectory of mortality trends for the last three forecast periods (S7 and S8 Figs), suggesting that the actual death counts might not be decreasing in Mexico City as seen with Mexico. Based on the mortality data, the observed decline or stability in death predictions could likely reflect the false slowing down of the pandemic in Mexico City [103]. Moreover, insufficient testing can also result in an inaccurate trajectory of the COVID-19 mortality curve [105].

The cumulative comparison of deaths in Mexico and Mexico City indicates that in general, the Richards model has under-performed in predicting the actual death counts with much wider uncertainty around the mean death estimates. The Richards model has also failed to capture the early sub-exponential growth dynamics of the mortality curve. The cumulative death counts obtained from the flexible sub-epidemic model closely approximate the total mean death counts obtained from the three IHME modeling scenarios. Whereas the GLM slightly under predicts the cumulative death counts (Figs 6 and 7). Another competing model, the COVID-19 predictions model projects 87,151 deaths (95% PI: [84,414, 91,883]) for Mexico as of October 27, 2020 (last forecasting phase), an estimate that closely approximates the estimate obtained from the GLM model (between 77,258–93,454 deaths) [106].

The three phenomenological models (GLM, Richards, sub-epidemic wave model) used in this study generally provide good fits to the mortality curves based on the residuals. However, the Richards model is unable to capture the early sub-exponential dynamics of the mortality curve. These phenomenological models are particularly valuable for providing rapid predictions of the epidemics in complex scenarios that can be used for real-time preparedness since these models do not require specific disease transmission processes to account for the interventions. Since these models do not explicitly account for behavioral changes, the results should be interpreted with caution. Importantly, since the mortality curves employed in this study are reported according to the date of reporting, they are likely influenced by variation in the testing rates and related factors including the case fatality rates. Further, delays in reporting of deaths due to the magnitude of the epidemic could also influence our predictions. Moreover, using the reporting date is not ideal due to the time difference between the date of death and the reporting date of death, which at a given moment can provide a false impression of the ongoing circumstances.

Our study is not exempt from limitations. First, the IHME (current projections, mandated mask, and worst-case scenario) model utilized has been revised multiple times over the course of the pandemic and differs substantially in methodology, assumptions, range of predictions, and quantities estimated. Second, the IHME has been irregular in publishing the downloadable estimates online for some periods. Third, we model the death estimates by date of reporting rather than by the date of death. Lastly, the unpredictable social component of the epidemic on the ground is also a limiting factor for the study as we do not know the ground truth mortality pattern when the forecasts are generated.

In conclusion, the reproduction number has been fluctuating around ~1.0 since the end of July-end of September 2020, indicating sustained virus transmission in the region. Simultaneously, the country has seen much lower mobility reduction and mixed compliance with stay-at-home orders contributing towards the virus transmission in the country. Moreover, the spatial analysis indicates that states like Mexico, Michoacán, Morelos, Nuevo Leon, Baja California require stronger public health strategies to contain the rising patterns in epidemic growth rates. The GLM and sub-epidemic model applied to mortality data in Mexico provide reasonable estimates for short-term projections in near real-time. While the GLM and Richards models predict that the COVID-19 outbreak in Mexico and Mexico City may be on a sustained decline, the sub-epidemic and IHME model predict a stabilization of daily deaths. However, the forecasts need to be interpreted with caution given the dynamic implementation and lifting of the social distancing measures.

## Supporting information

S1 File (DOCX)

S1 Fig COVID-19 deaths forecasts using daily deaths, GLM model, Mexico: 30-days ahead forecasts based on the Generalized Logistic Growth model (GLM) calibrated using an increasing amount of daily death data (blue circles): 107, 114, 120, 128, 136, 151, 156, 164, 172, 179, 185, 193, 193 epidemic days. The vertical dashed line indicates the end of the calibration period and start of the forecasting period. The mean (solid red line) and 95% PIs (dashed red lines) of the model fit and forecast are shown.
(TIF)

S2 Fig COVID-19 death forecasts using daily deaths, Richards model, Mexico: 30-days ahead forecasts based on the Richards model calibrated using an increasing amount of daily death data (blue circles): 107, 114, 120, 128, 136, 151, 156, 164, 172, 179, 185, 193, 193 epidemic days. The vertical dashed line indicates the end of the calibration period and start of the forecasting period. The mean (solid red line) and 95% PIs (dashed red lines) of the model fit and forecast are shown.
(TIF)

S3 Fig COVID-19 death forecasts using daily deaths, GLM model, Mexico City: 30-days ahead forecasts based on the GLM model calibrated using an increasing amount of daily death data (blue circles): 107, 114, 120, 128, 136, 151, 156, 164, 172, 179, 185, 193, 193 epidemic days. The vertical dashed line indicates the end of the calibration period and start of the forecasting period. The mean (solid red line) and 95% PIs (dashed red lines) of the model fit and forecast are shown.
(TIF)

S4 Fig COVID-19 death forecasts using daily deaths, Richards model, Mexico City: 30-days ahead forecasts based on the Richards model calibrated using an increasing amount of daily death data (blue circles): 107, 114, 120, 128, 136, 151, 156, 164, 172, 179, 185, 193, 193 epidemic days. The vertical dashed line indicates the end of the calibration period and start of the forecasting period. The mean (solid red line) and 95% PIs (dashed red lines) of the model fit and forecast are shown.
(TIF)

S5 Fig COVID-19 death forecasts using daily deaths, IHME model, Mexico: 30-days ahead forecasts based on the IHME model calibrated using an increasing amount of daily death data (blue circles): 107, 114, 120, 128, 136, 151, 156, 164, 172, 179, 185, 193, 193 epidemic days. The vertical dashed line indicates the end of the calibration period and start of the forecasting period. The mean (solid red line) and 95% PIs (dashed red lines) of the model fit and forecast are shown.
(TIF)

S6 Fig COVID-19 death forecasts using daily deaths, sub-epidemic wave model, Mexico: 30-days ahead forecasts based on the sub-epidemic wave model calibrated using an increasing amount of daily death data (blue circles): 107, 114, 120, 128, 136, 151, 156, 164, 172, 179, 185, 193, 193 epidemic days. The vertical dashed line indicates the end of the calibration period and start of the forecasting period. The mean (solid red line) and 95% PIs (dashed red lines) of the model fit and forecast are shown.
(TIF)

S7 Fig COVID-19 death forecasts using daily deaths, IHME model, Mexico City: 30-days ahead forecasts based on the IHME model calibrated using an increasing amount of daily death data (blue circles): 107, 114, 120, 128, 136, 151, 156, 164, 172, 179, 185, 193, 193 epidemic days. The vertical dashed line indicates the end of the calibration period and start of the forecasting period. The mean (solid red line) and 95% PIs (dashed red lines) of the model fit and forecast are shown.
(TIF)

S8 Fig COVID-19 death forecasts using daily deaths, sub-epidemic wave model, Mexico City: 30-days ahead forecasts based on the sub-epidemic wave model calibrated using an increasing amount of daily death data (blue circles): 107, 114, 120, 128, 136, 151, 156, 164, 172, 179, 185, 193, 193 epidemic days. The vertical dashed line indicates the end of the calibration period and start of the forecasting period. The mean (solid red line) and 95% PIs (dashed red lines) of the model fit and forecast are shown.
(TIF)

S9 Fig COVID-19 deaths forecasts using cumulative deaths, GLM model, Mexico: 30-days ahead forecasts based on the Generalized Logistic Growth model (GLM) calibrated using an increasing amount of cumulative death data (blue circles). The vertical dashed line indicates the end of the calibration period and start of the forecasting period. The mean (solid red line) and 95% PIs (dashed red lines) of the model fit and forecast are shown.
(TIF)

S10 Fig COVID-19 death forecasts using cumulative deaths, IHME model, Mexico: 30-day ahead forecasts based on the IHME model calibrated using cumulative death data (blue circles). The vertical dashed line indicates the end of the calibration period and start of the forecasting period. The mean (solid red line) and 95% PIs (dashed red lines) of the model fit and forecast are shown.
(TIF)

S11 Fig COVID-19 death forecasts using cumulative deaths, Richards model, Mexico: 30-day ahead forecasts based on the Richards model calibrated using cumulative death data (blue circles). The vertical dashed line indicates the end of the calibration period and start of the forecasting period. The mean (solid red line) and 95% PIs (dashed red lines) of the model fit and forecast are shown.
(TIF)

S12 Fig COVID-19 death forecasts using cumulative deaths, sub-epidemic wave model, Mexico: 30-day ahead forecasts based on the Sub-epidemic wave model calibrated using cumulative death data (blue circles). The vertical dashed line indicates the end of the calibration period and start of the forecasting period. The mean (solid red line) and 95% PIs (dashed red lines) of the model fit and forecast are shown.
(TIF)

S13 Fig COVID-19 deaths forecasts using cumulative deaths, GLM model, Mexico City: 30-day ahead forecasts based on the Generalized Logistic Growth model (GLM) calibrated using cumulative death data (blue circles). The vertical dashed line indicates the end of the calibration period and start of the forecasting period. The mean (solid red line) and 95% PIs (dashed red lines) of the model fit and forecast are shown.
(TIF)

S14 Fig COVID-19 death forecasts using cumulative deaths, IHME model, Mexico City: 30-day ahead forecasts based on the IHME model calibrated using cumulative death data (blue circles). The vertical dashed line indicates the end of the calibration period and start of the forecasting period. The mean (solid red line) and 95% PIs (dashed red lines) of the model fit and forecast are shown.
(TIF)

S15 Fig COVID-19 death forecasts using cumulative deaths, Richards model, Mexico City: 30-day ahead forecasts based on the Richards model calibrated using cumulative death data (blue circles). The vertical dashed line indicates the end of the calibration period and start of the forecasting period. The mean (solid red line) and 95% PIs (dashed red lines) of the model fit and forecast are shown.
(TIF)

S16 Fig COVID-19 death forecasts using cumulative deaths, sub-epidemic wave model, Mexico City: 30-day ahead forecasts based on the Sub-epidemic wave model calibrated using cumulative death data (blue circles). The vertical dashed line indicates the end of the calibration period and start of the forecasting period. The mean (solid red line) and 95% PIs (dashed red lines) of the model fit and forecast are shown.
(TIF)

S17 Fig Pre-processing COVID-19 data into incidence rate functions. From left to right: original lab-confirmed COVID-19 cases, curve of daily new cases, smoothed and scaled rate curves, average of rate curves before scaling and smothing.
(TIF)

S18 Fig Clustering of states according to the shapes of their rate curves. The largest cluster–cluster 1 –is shown in green while the smallest cluster–cluster 3 –is shown in the black. One can see that states with similar shapes of rates curves are geographically close to each other.
(TIF)

S19 Fig Average shapes of the COVID-19 incidence rate curves, along with a one standard-deviation band around the average, in each of the clusters. (TIF)

S20 Fig Cluster averages and the overall average. These averages represent the four dominant patterns of incidence rates observed across all states.
(TIF)

S21 Fig Total number of COVID-19 cases as of December 5, 2020. (TIF)

S22 Fig COVID-19 epi-curve overlaid by the curve of stay-at-home orders tweets. Blue line indicates the number of cases by dates of onset and the orange line indicates the number of tweets referring to the stay-at-home orders.
(TIF)
