# Supplementary figures and images for: Transmission dynamics and forecasts of the COVID-19 pandemic in Mexico, March-December 2020
Source: PLoS One. 2021 Jul 21;16(7):e0254826. doi: 10.1371/journal.pone.0254826 (PMC8294497; doi:10.1371/journal.pone.0254826)

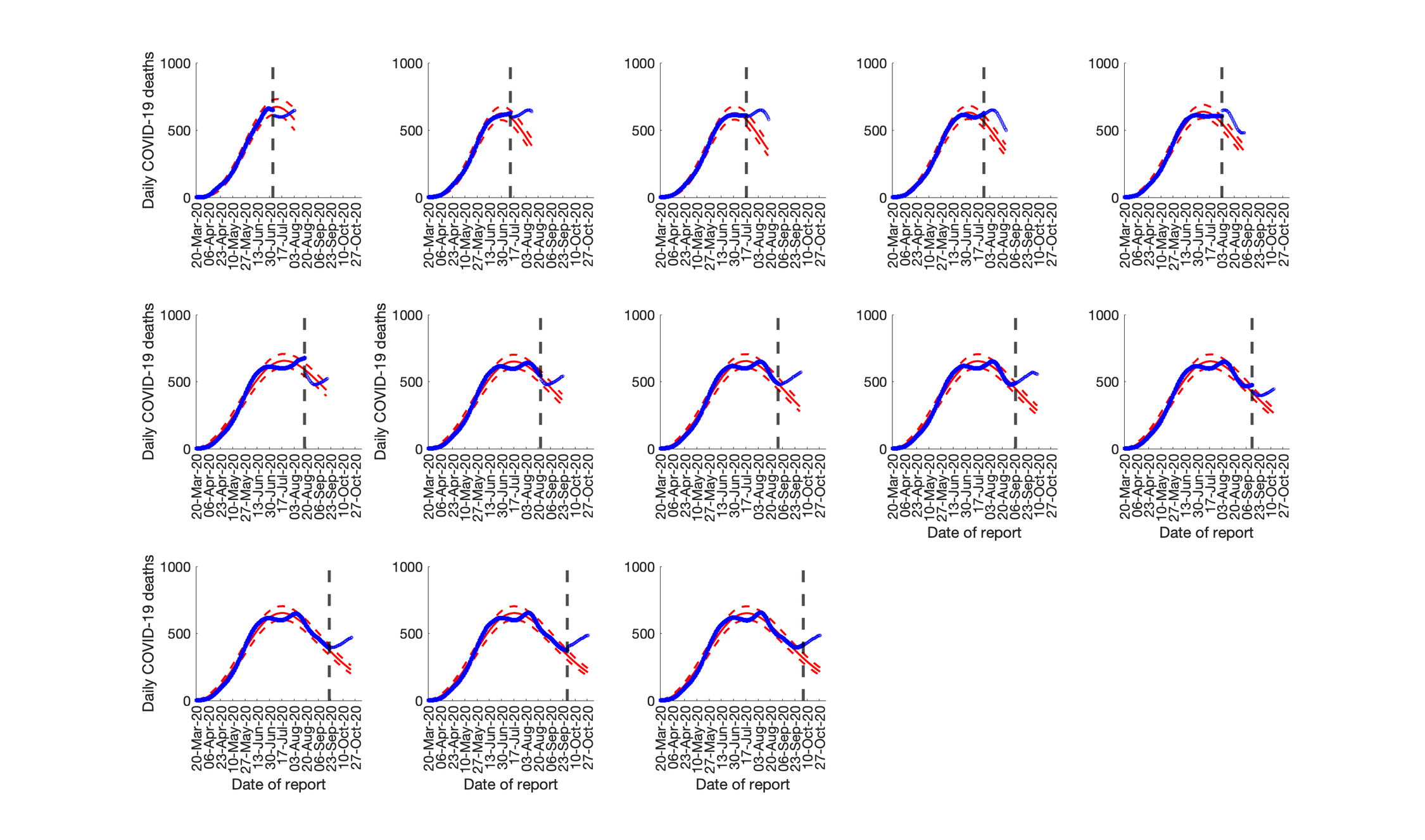

Supplement: S1 Fig — The vertical dashed line indicates the end of the calibration period and start of the forecasting period. The mean (solid red line) and 95% PIs (dashed red lines) of the model fit and forecast are shown. (TIF) [file pone.0254826.s002.tif]

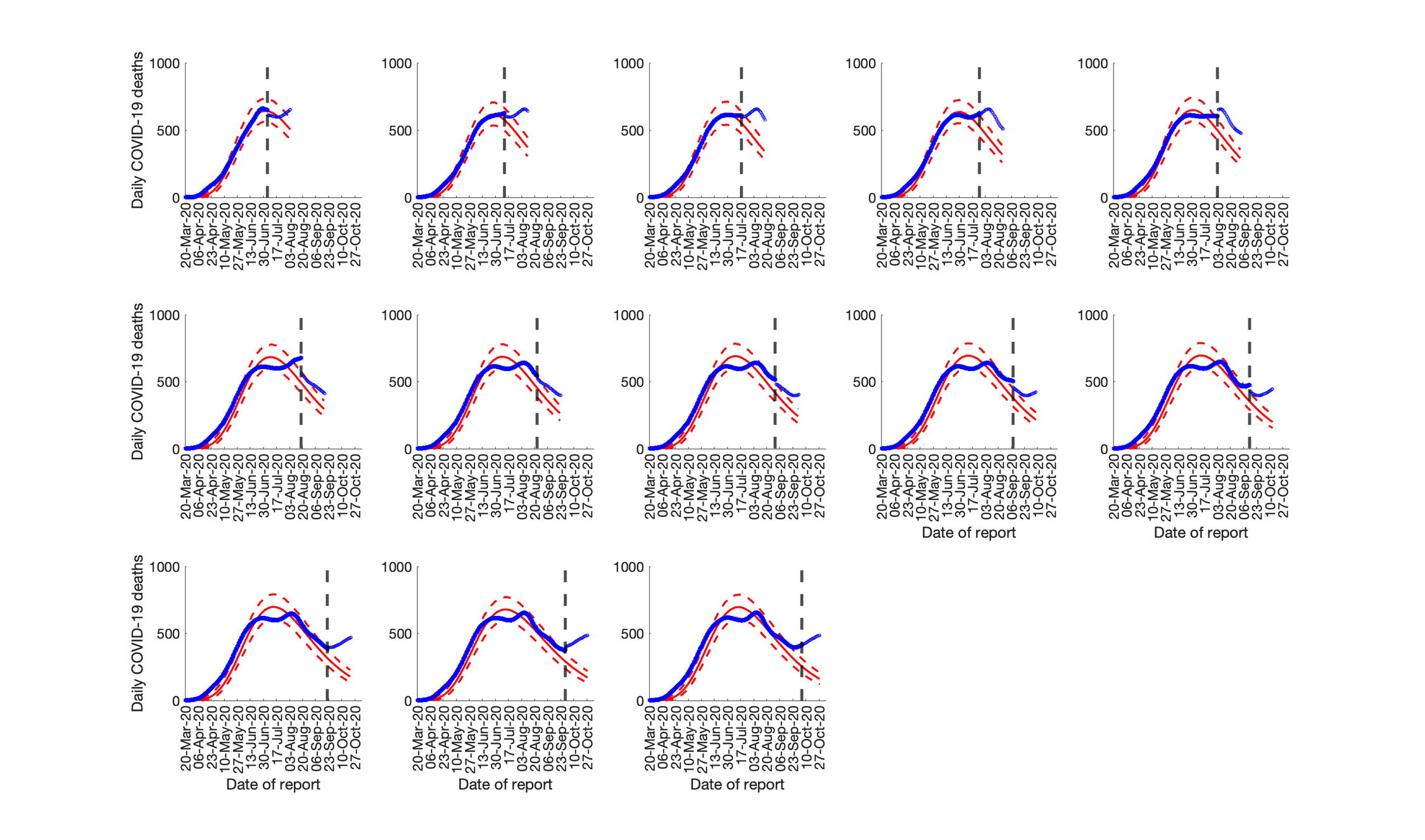

Supplement: S2 Fig — The vertical dashed line indicates the end of the calibration period and start of the forecasting period. The mean (solid red line) and 95% PIs (dashed red lines) of the model fit and forecast are shown. (TIF) [file pone.0254826.s003.tif]

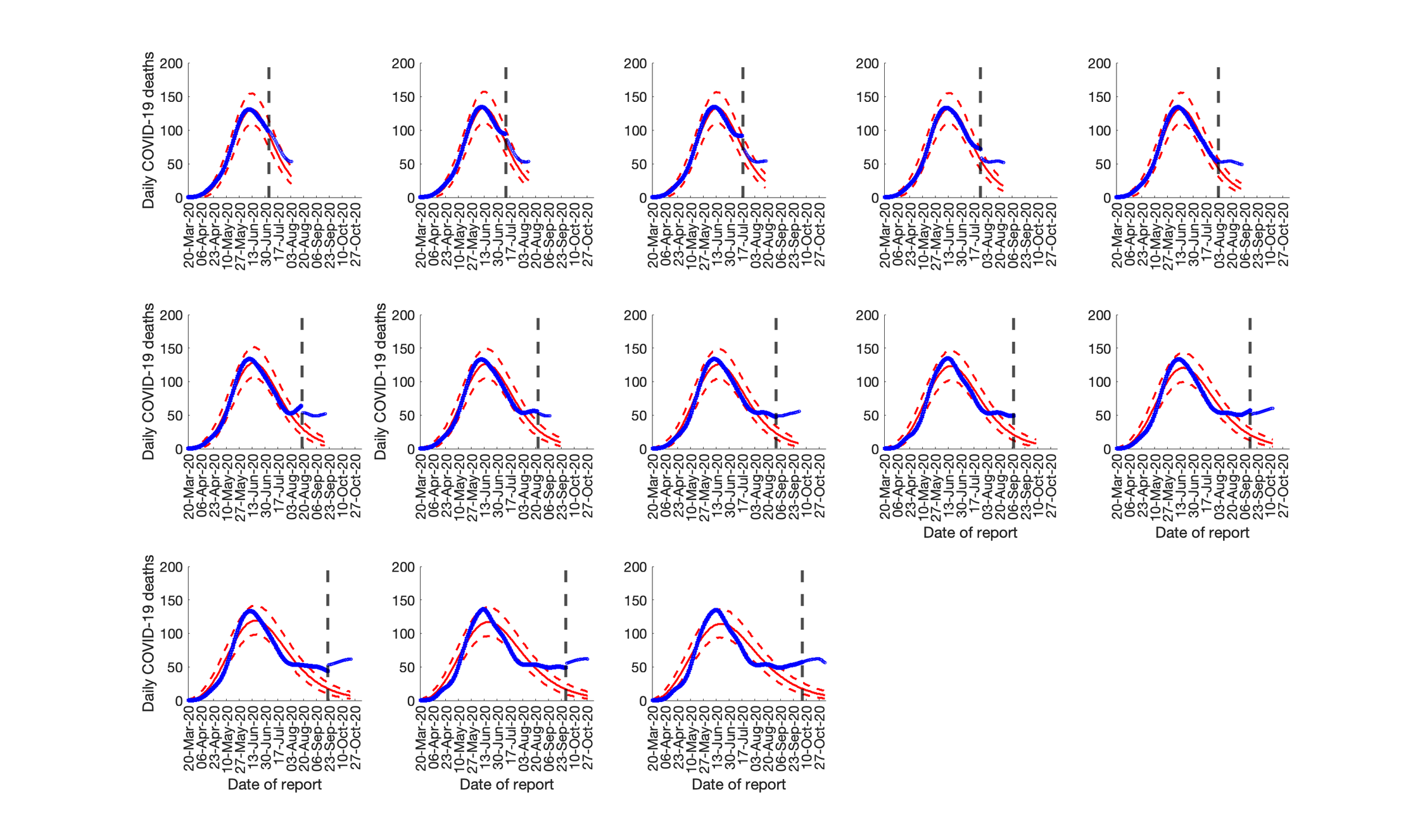

Supplement: S3 Fig — The vertical dashed line indicates the end of the calibration period and start of the forecasting period. The mean (solid red line) and 95% PIs (dashed red lines) of the model fit and forecast are shown. (TIF) [file pone.0254826.s004.tif]

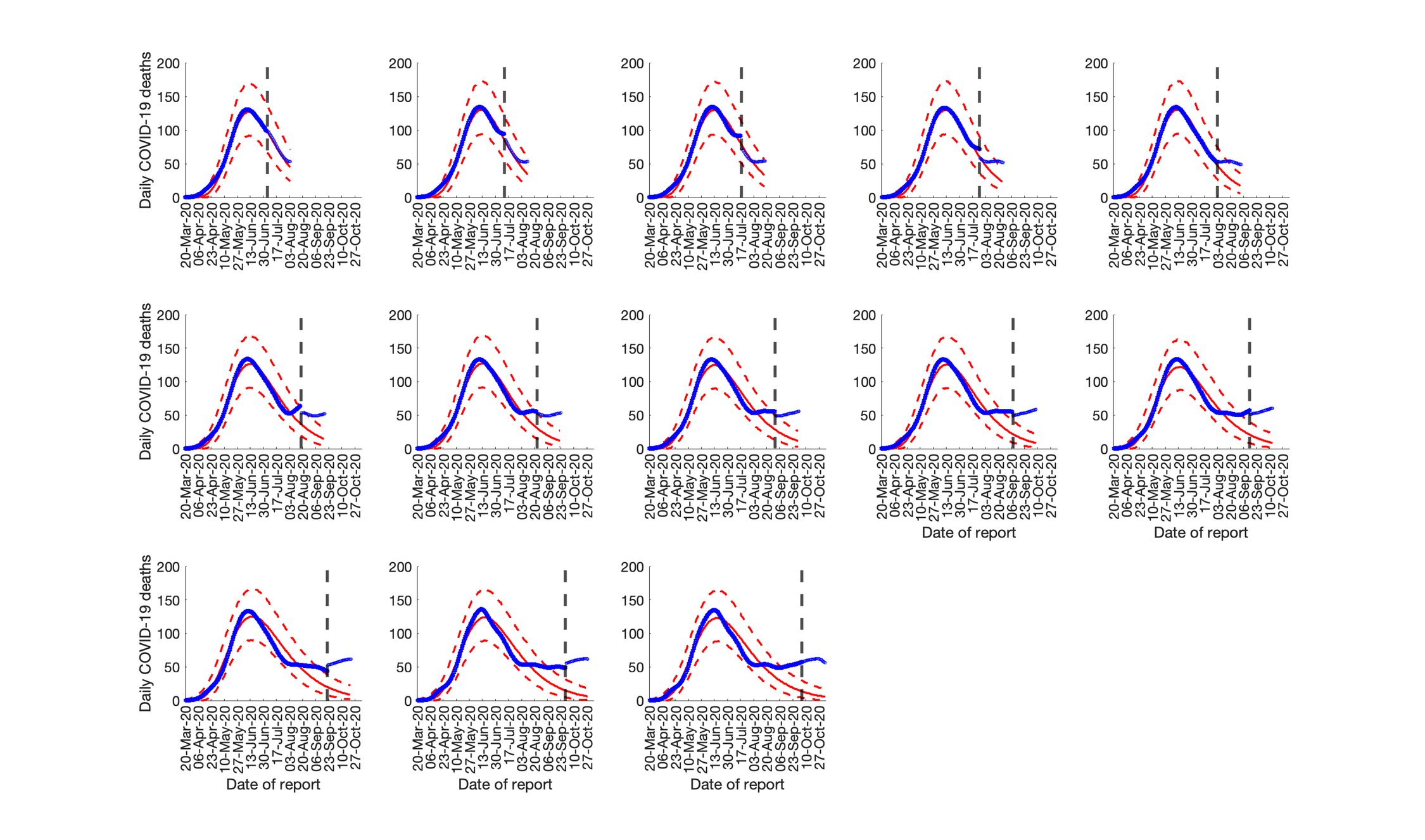

Supplement: S4 Fig — The vertical dashed line indicates the end of the calibration period and start of the forecasting period. The mean (solid red line) and 95% PIs (dashed red lines) of the model fit and forecast are shown. (TIF) [file pone.0254826.s005.tif]

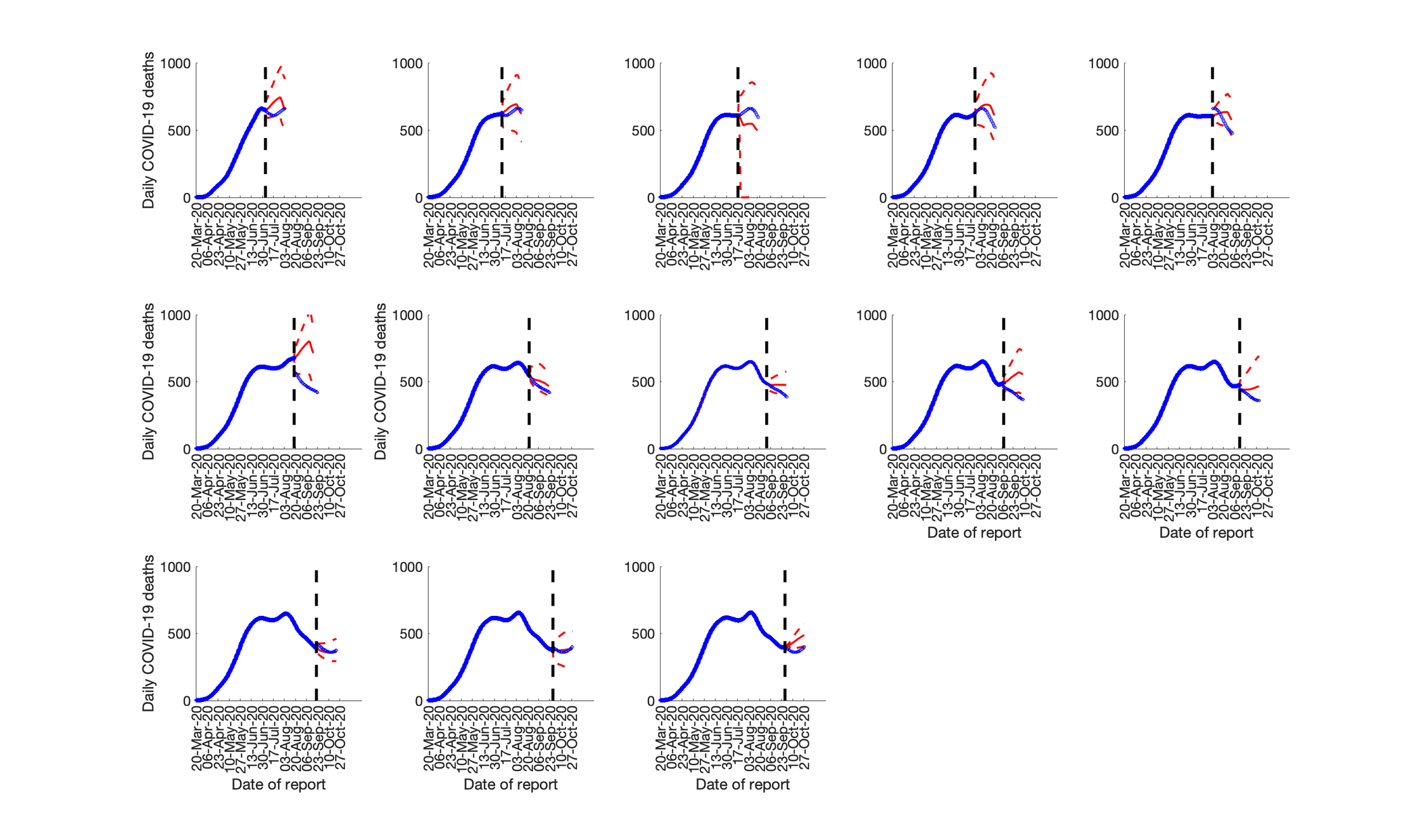

Supplement: S5 Fig — The vertical dashed line indicates the end of the calibration period and start of the forecasting period. The mean (solid red line) and 95% PIs (dashed red lines) of the model fit and forecast are shown. (TIF) [file pone.0254826.s006.tif]

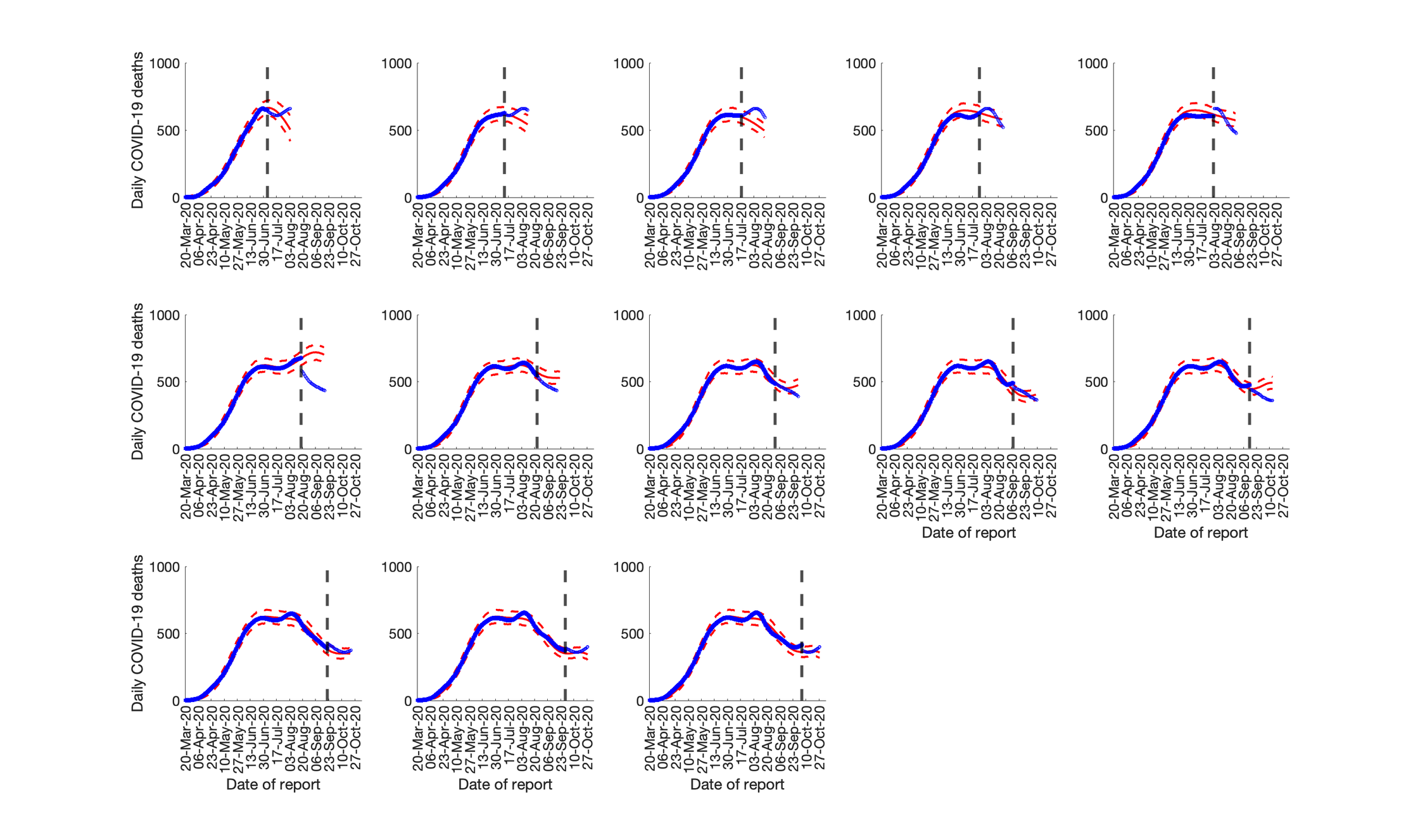

Supplement: S6 Fig — The vertical dashed line indicates the end of the calibration period and start of the forecasting period. The mean (solid red line) and 95% PIs (dashed red lines) of the model fit and forecast are shown. (TIF) [file pone.0254826.s007.tif]

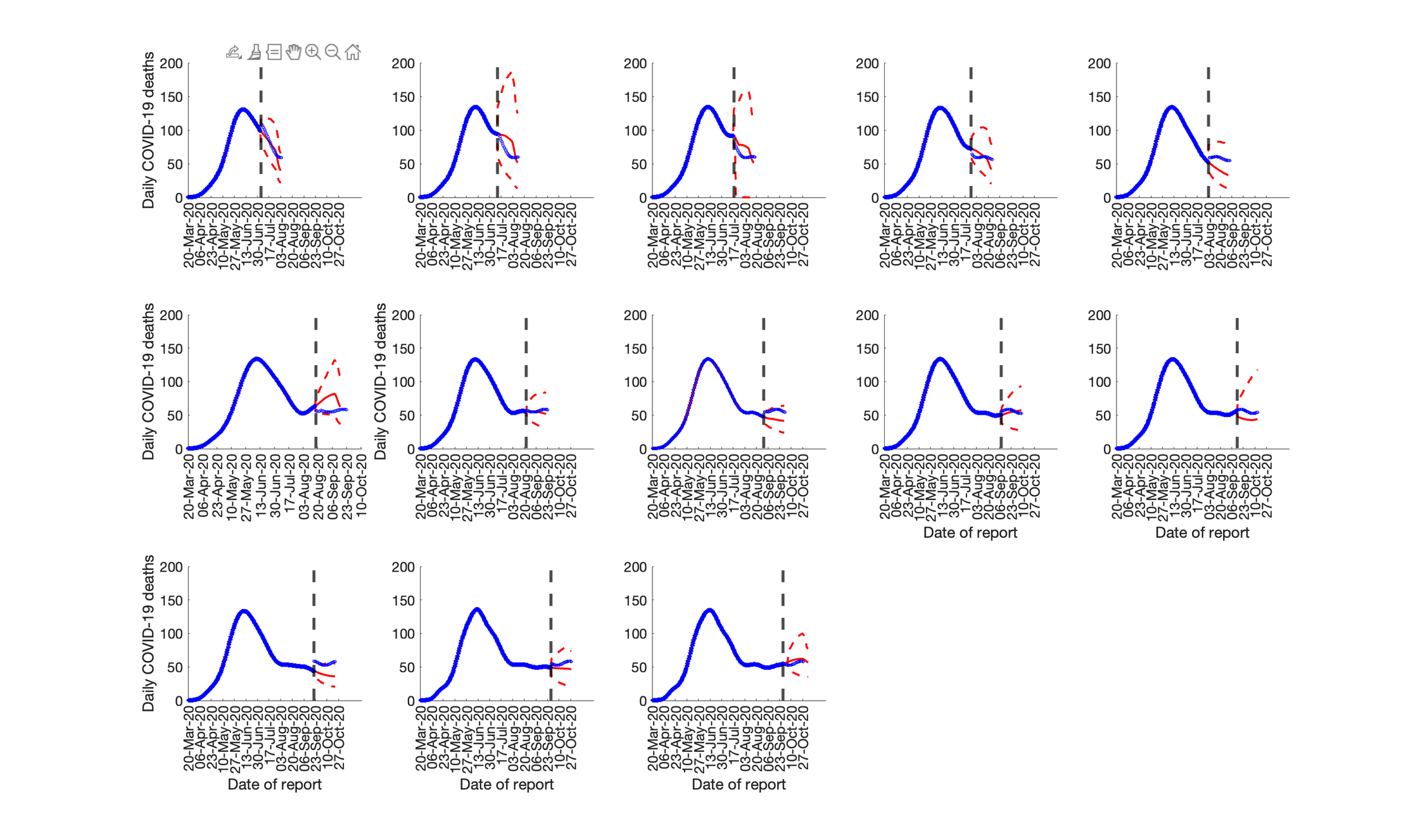

Supplement: S7 Fig — The vertical dashed line indicates the end of the calibration period and start of the forecasting period. The mean (solid red line) and 95% PIs (dashed red lines) of the model fit and forecast are shown. (TIF) [file pone.0254826.s008.tif]

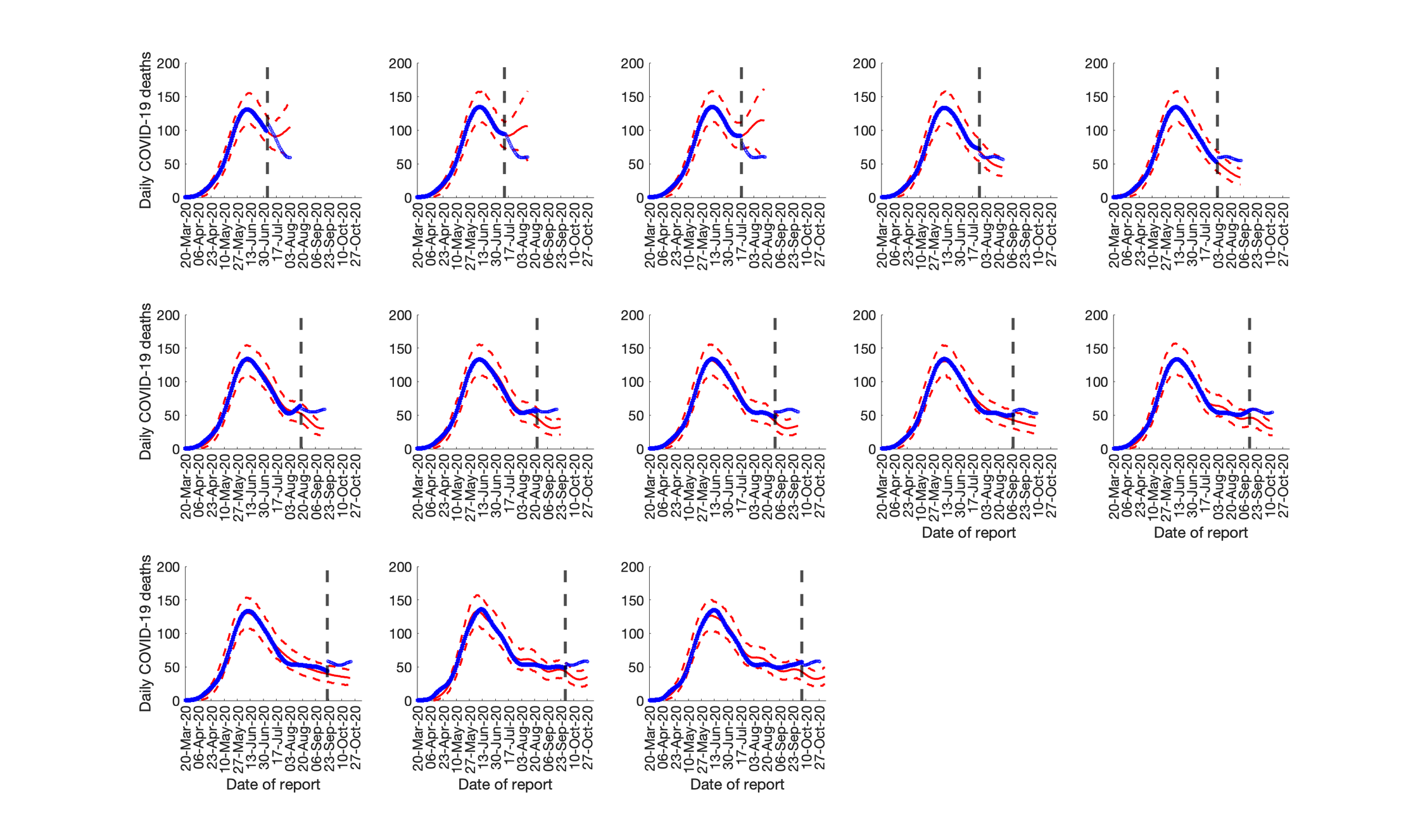

Supplement: S8 Fig — The vertical dashed line indicates the end of the calibration period and start of the forecasting period. The mean (solid red line) and 95% PIs (dashed red lines) of the model fit and forecast are shown. (TIF) [file pone.0254826.s009.tif]

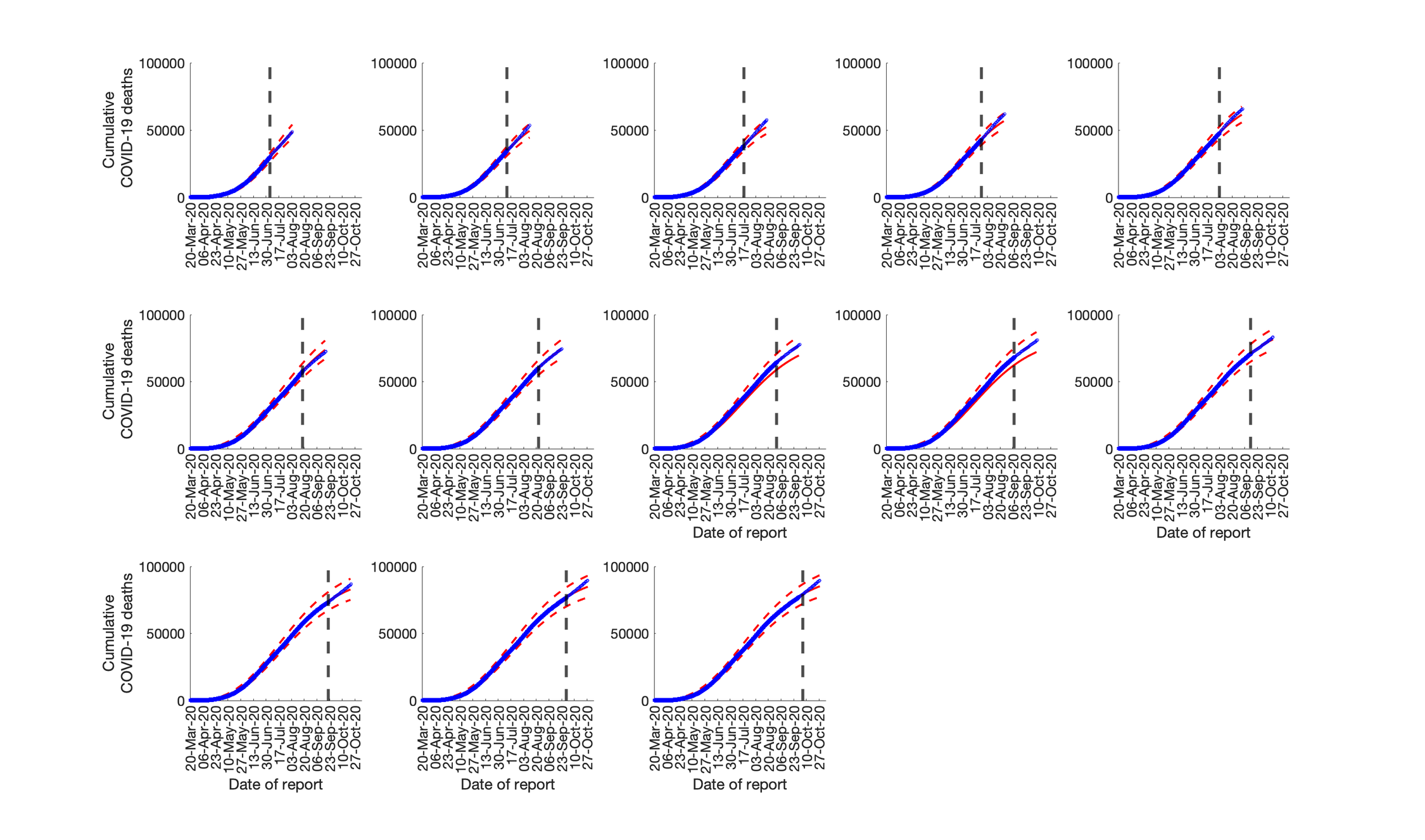

Supplement: S9 Fig — The vertical dashed line indicates the end of the calibration period and start of the forecasting period. The mean (solid red line) and 95% PIs (dashed red lines) of the model fit and forecast are shown. (TIF) [file pone.0254826.s010.tif]

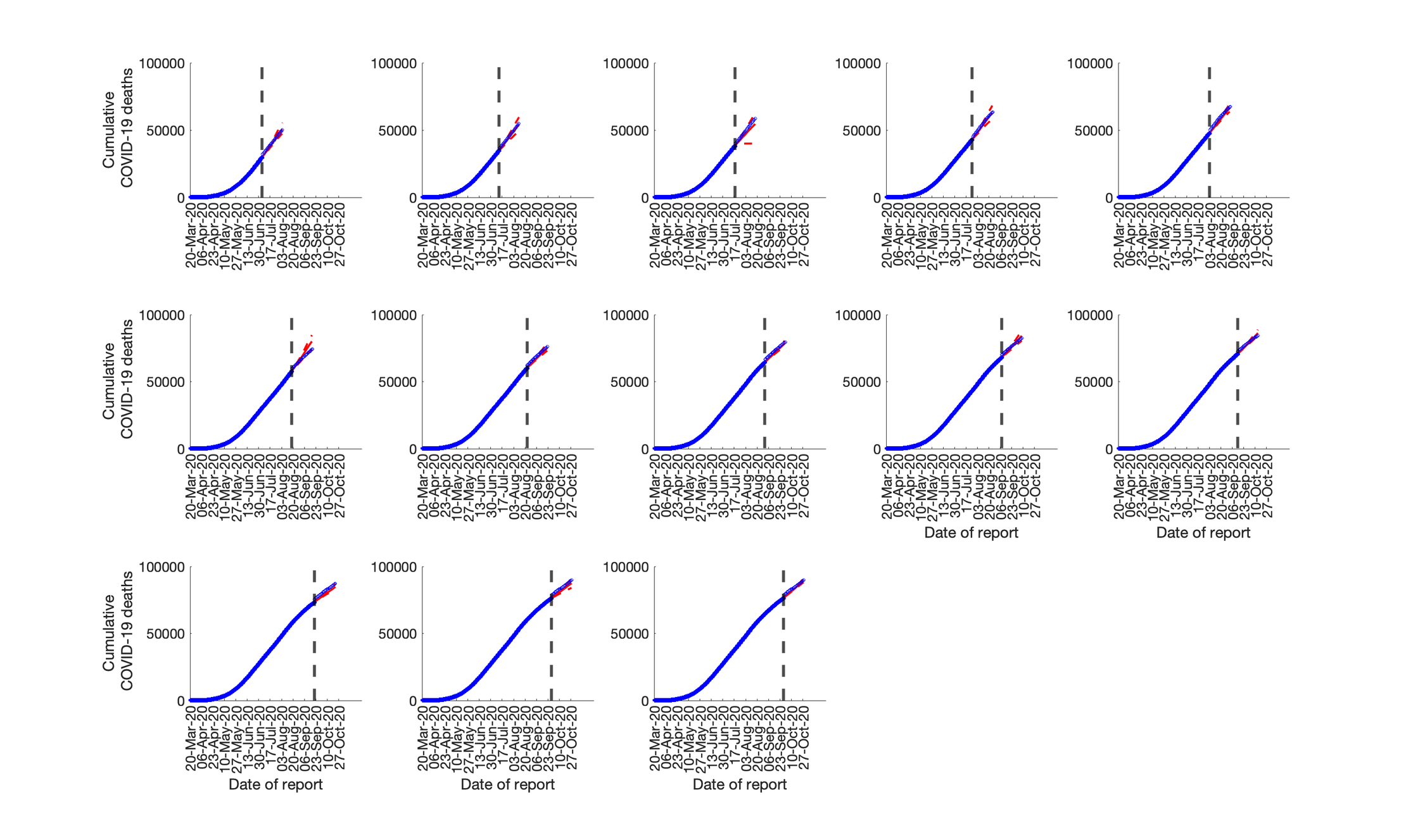

Supplement: S10 Fig — The vertical dashed line indicates the end of the calibration period and start of the forecasting period. The mean (solid red line) and 95% PIs (dashed red lines) of the model fit and forecast are shown. (TIF) [file pone.0254826.s011.tif]

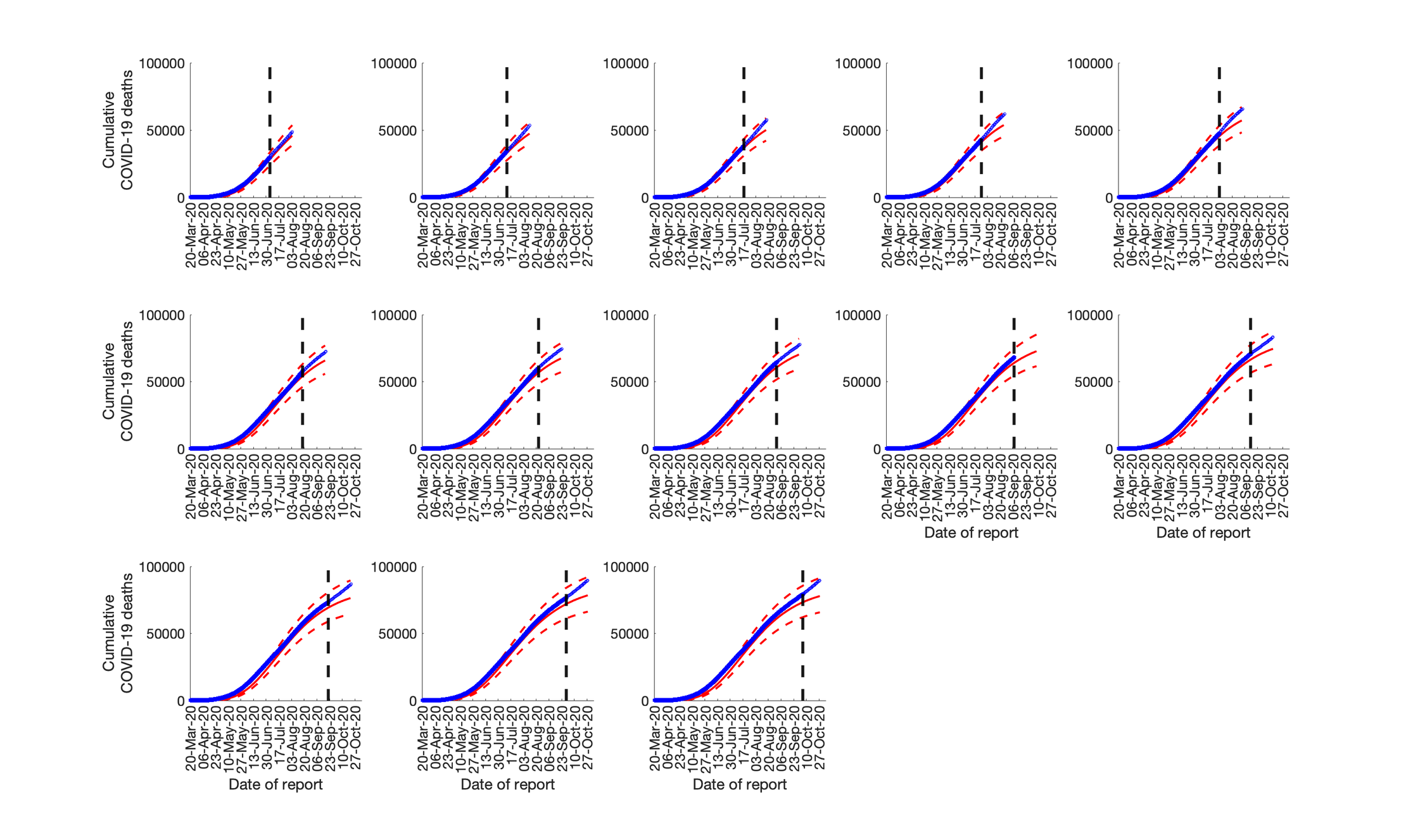

Supplement: S11 Fig — The vertical dashed line indicates the end of the calibration period and start of the forecasting period. The mean (solid red line) and 95% PIs (dashed red lines) of the model fit and forecast are shown. (TIF) [file pone.0254826.s012.tif]

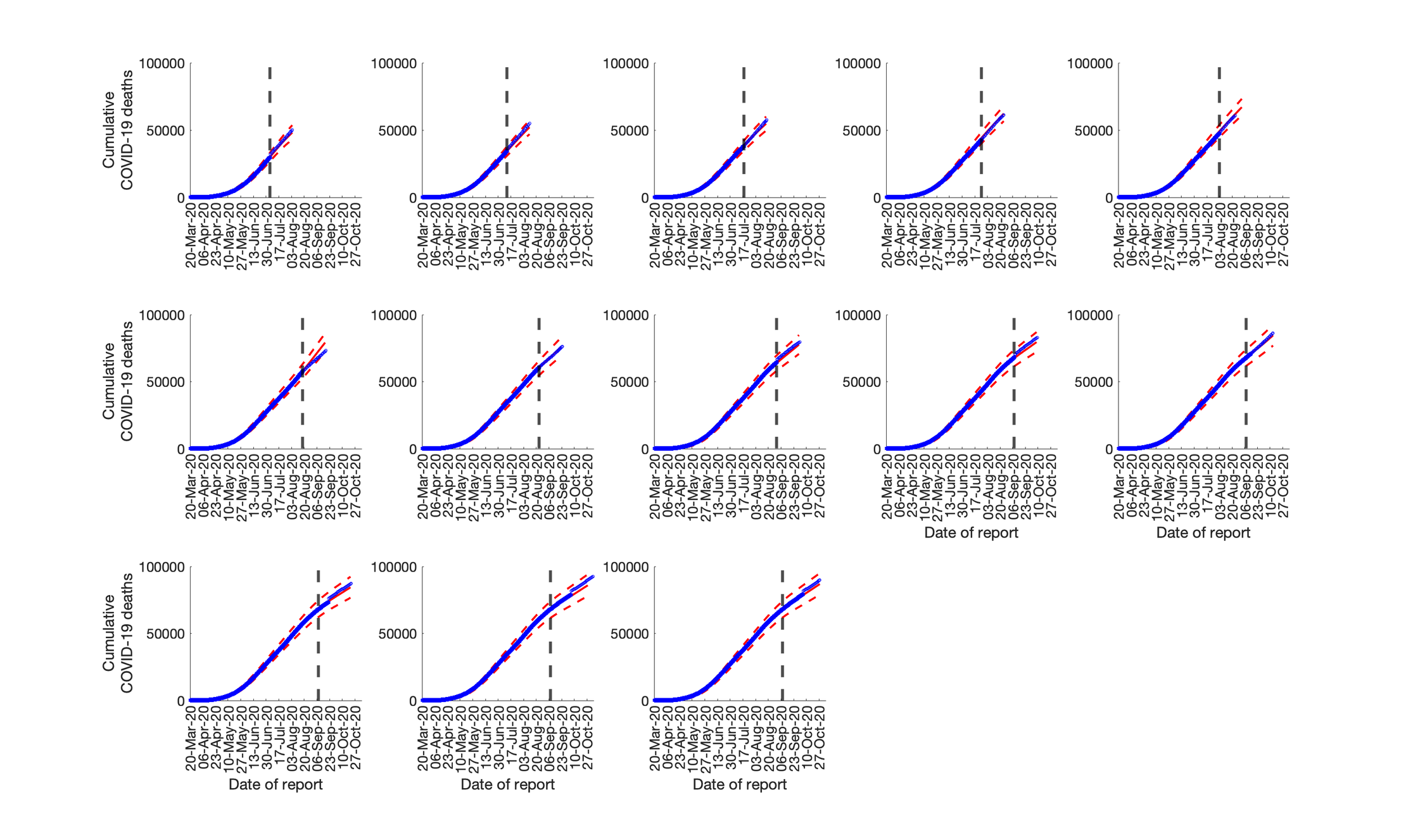

Supplement: S12 Fig — The vertical dashed line indicates the end of the calibration period and start of the forecasting period. The mean (solid red line) and 95% PIs (dashed red lines) of the model fit and forecast are shown. (TIF) [file pone.0254826.s013.tif]

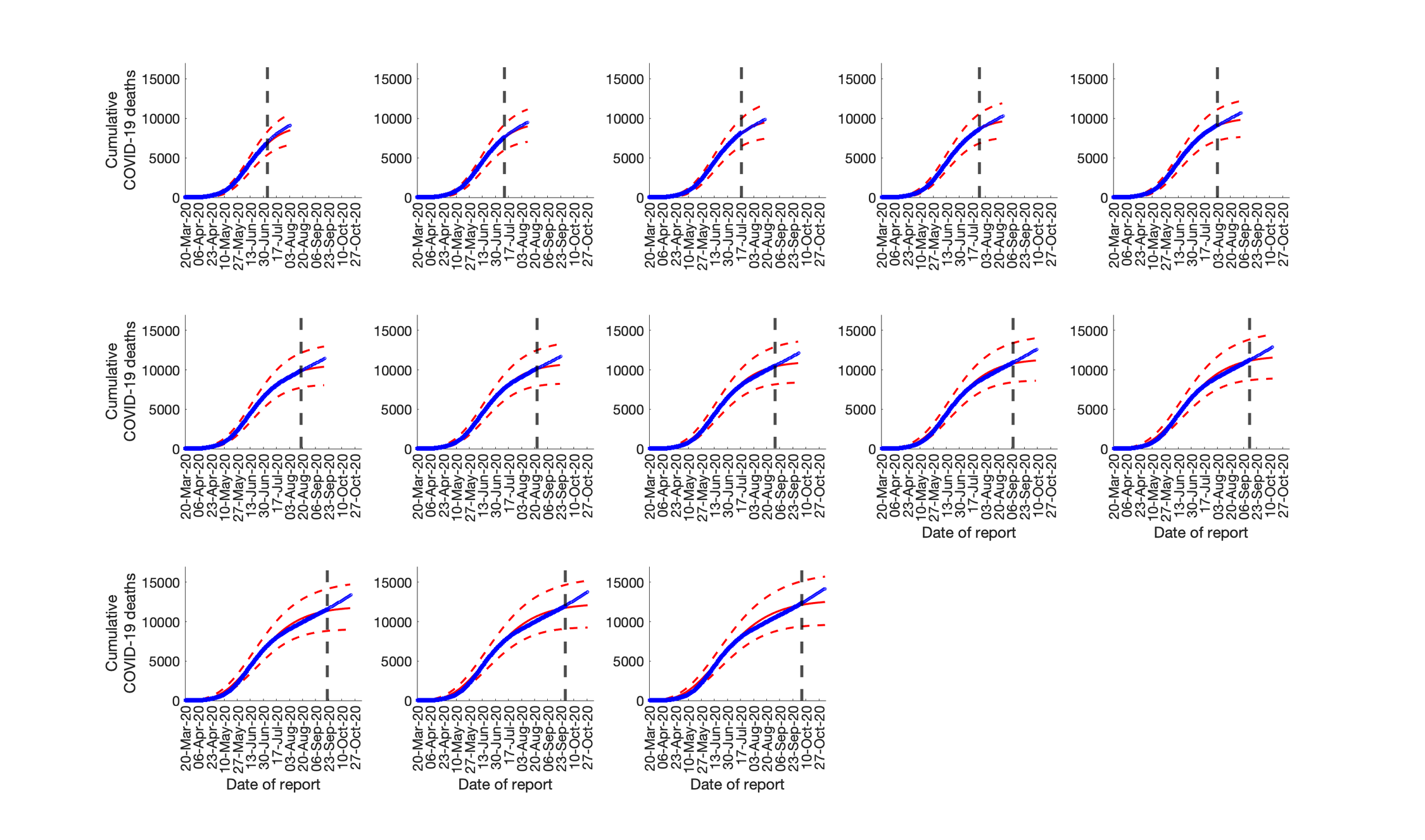

Supplement: S13 Fig — The vertical dashed line indicates the end of the calibration period and start of the forecasting period. The mean (solid red line) and 95% PIs (dashed red lines) of the model fit and forecast are shown. (TIF) [file pone.0254826.s014.tif]

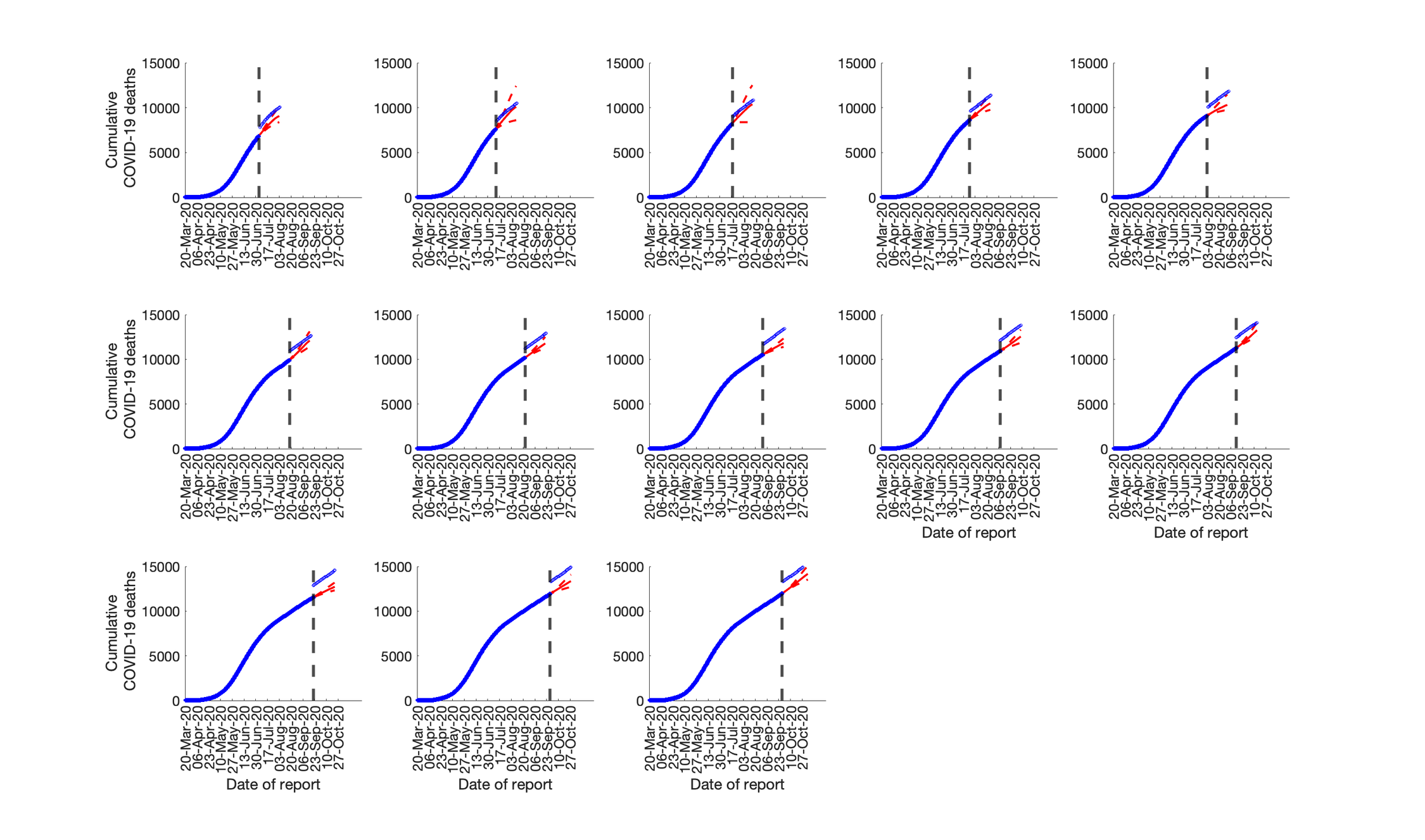

Supplement: S14 Fig — The vertical dashed line indicates the end of the calibration period and start of the forecasting period. The mean (solid red line) and 95% PIs (dashed red lines) of the model fit and forecast are shown. (TIF) [file pone.0254826.s015.tif]

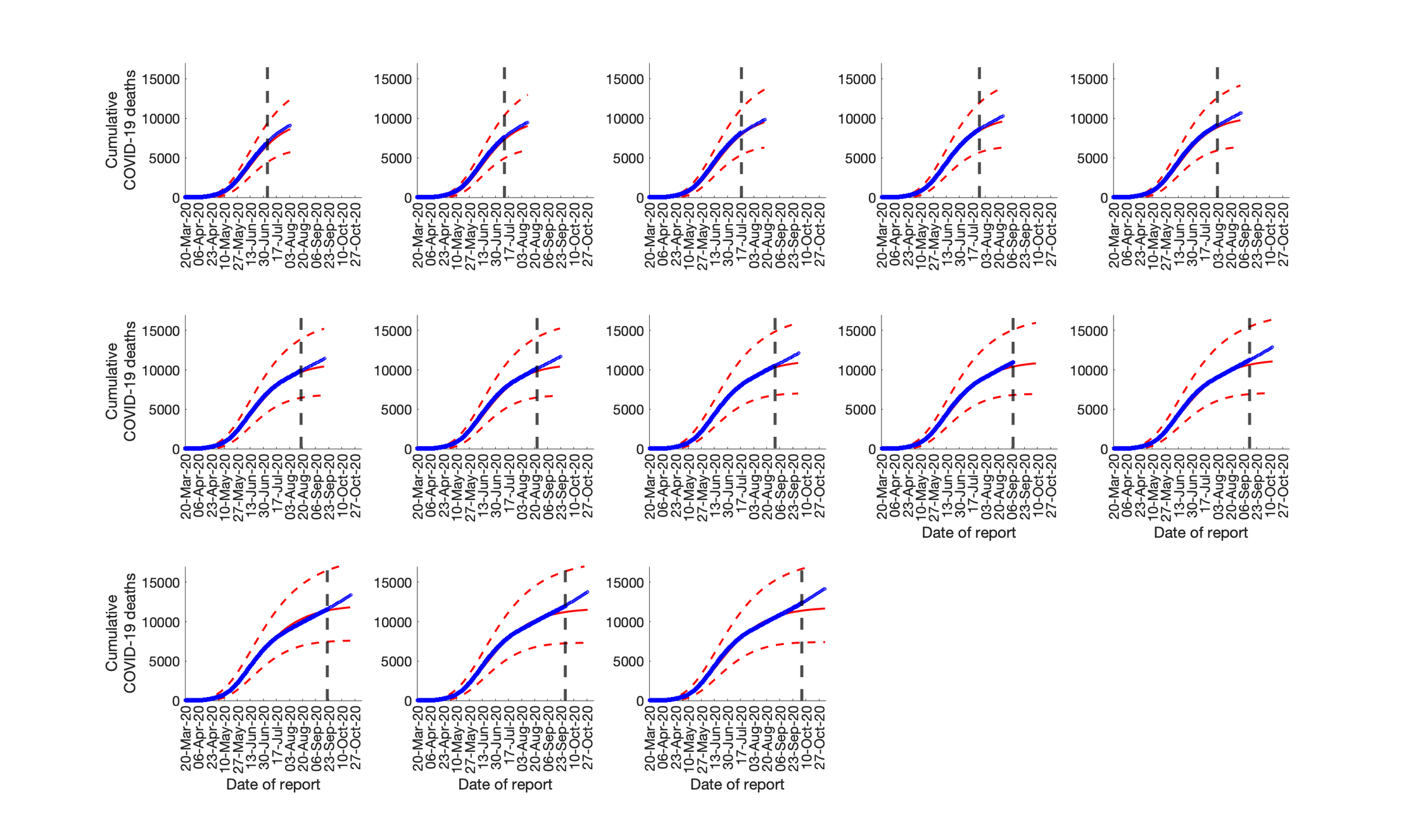

Supplement: S15 Fig — The vertical dashed line indicates the end of the calibration period and start of the forecasting period. The mean (solid red line) and 95% PIs (dashed red lines) of the model fit and forecast are shown. (TIF) [file pone.0254826.s016.tif]

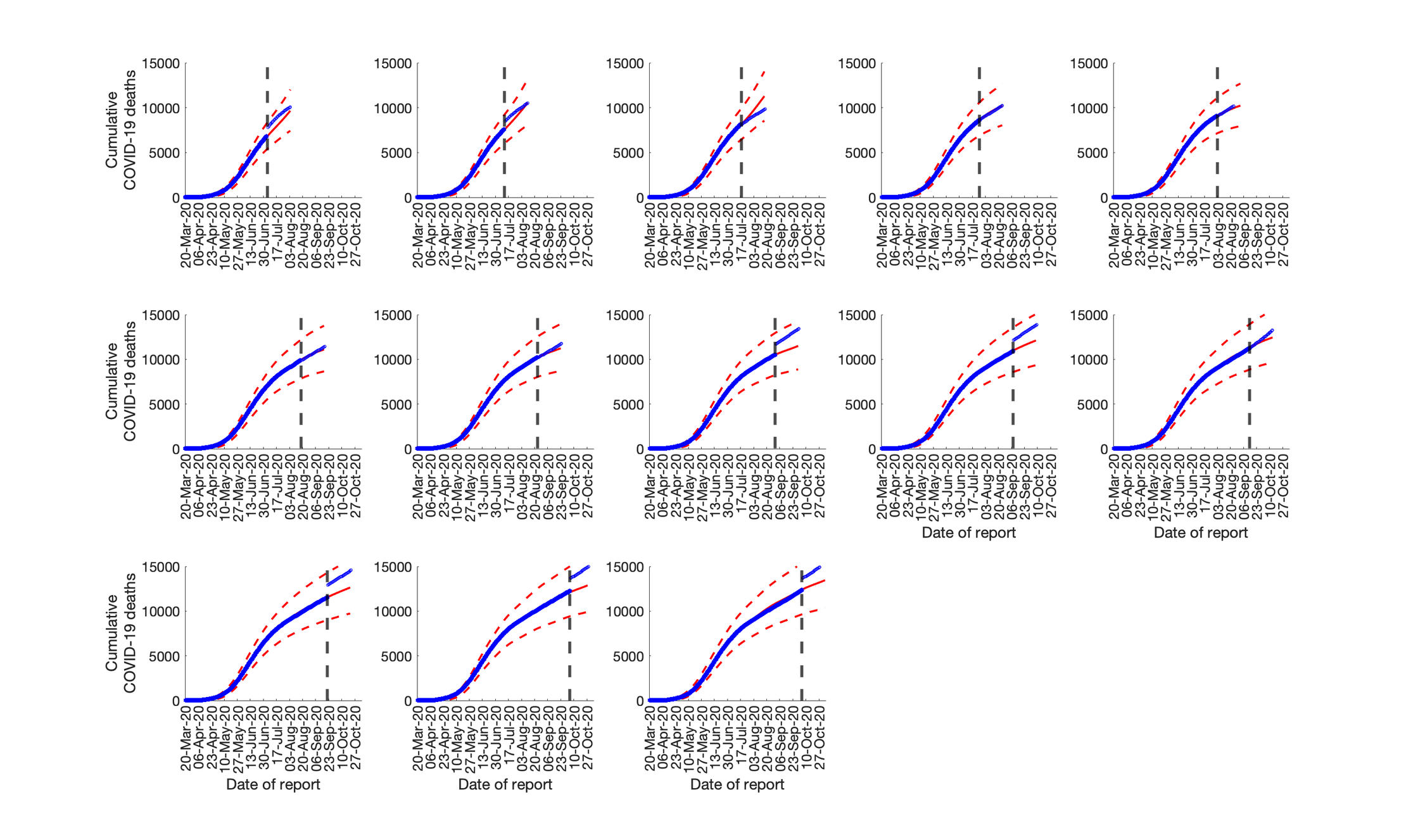

Supplement: S16 Fig — The vertical dashed line indicates the end of the calibration period and start of the forecasting period. The mean (solid red line) and 95% PIs (dashed red lines) of the model fit and forecast are shown. (TIF) [file pone.0254826.s017.tif]

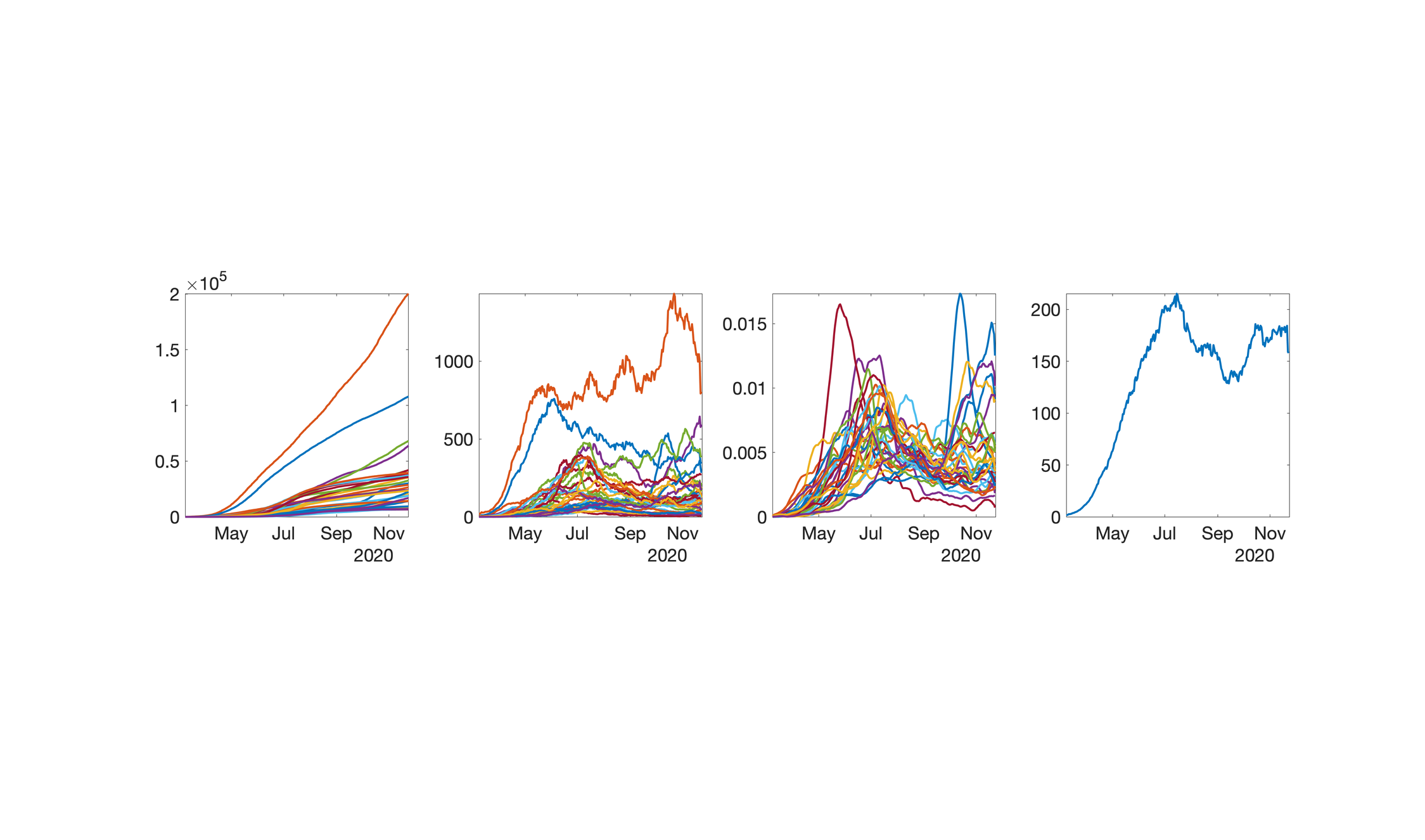

Supplement: S17 Fig — From left to right: original lab-confirmed COVID-19 cases, curve of daily new cases, smoothed and scaled rate curves, average of rate curves before scaling and smothing. (TIF) [file pone.0254826.s018.tif]

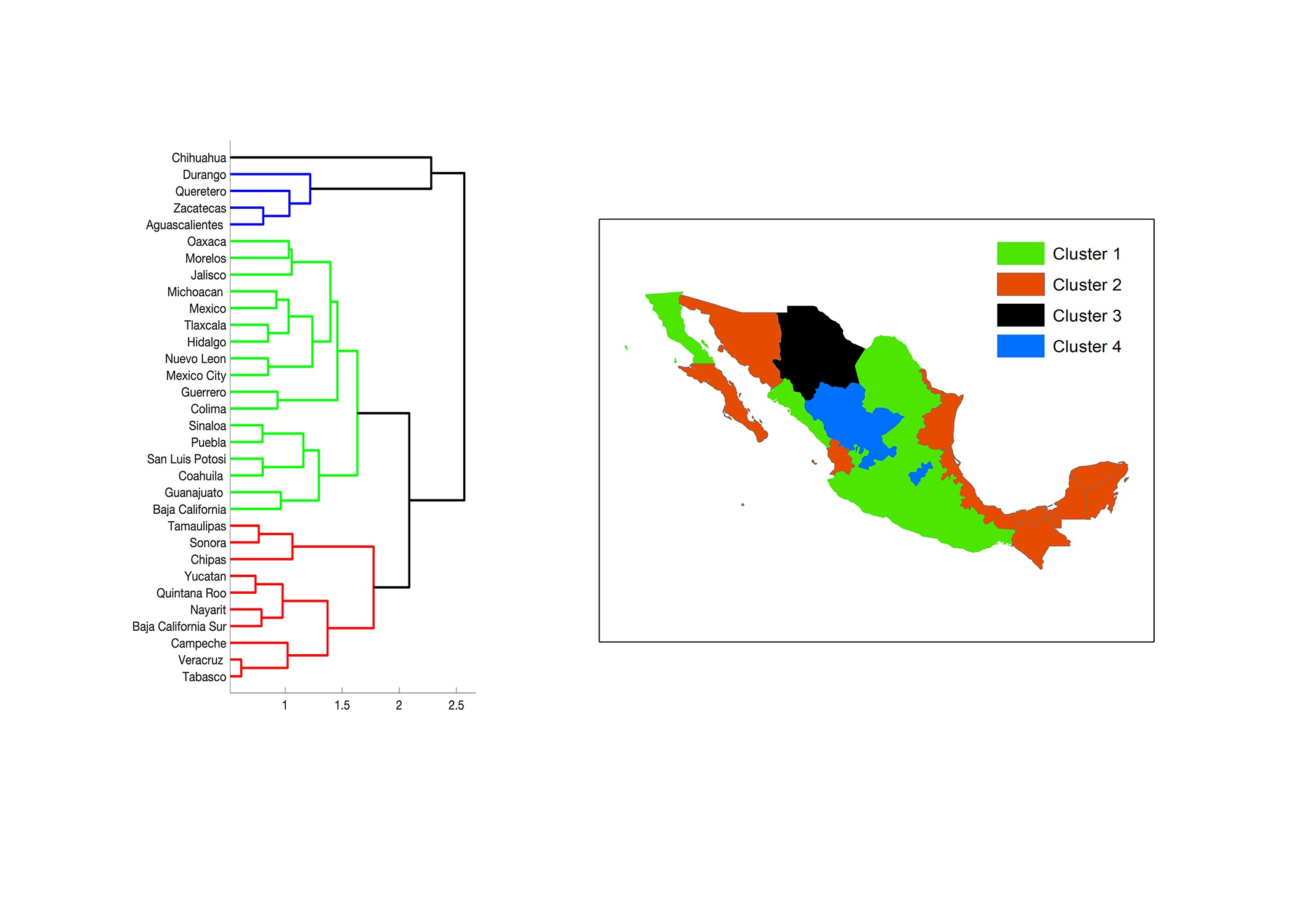

Supplement: S18 Fig — The largest cluster–cluster 1 –is shown in green while the smallest cluster–cluster 3 –is shown in the black. One can see that states with similar shapes of rates curves are geographically close to each other. (TIF) [file pone.0254826.s019.tif]

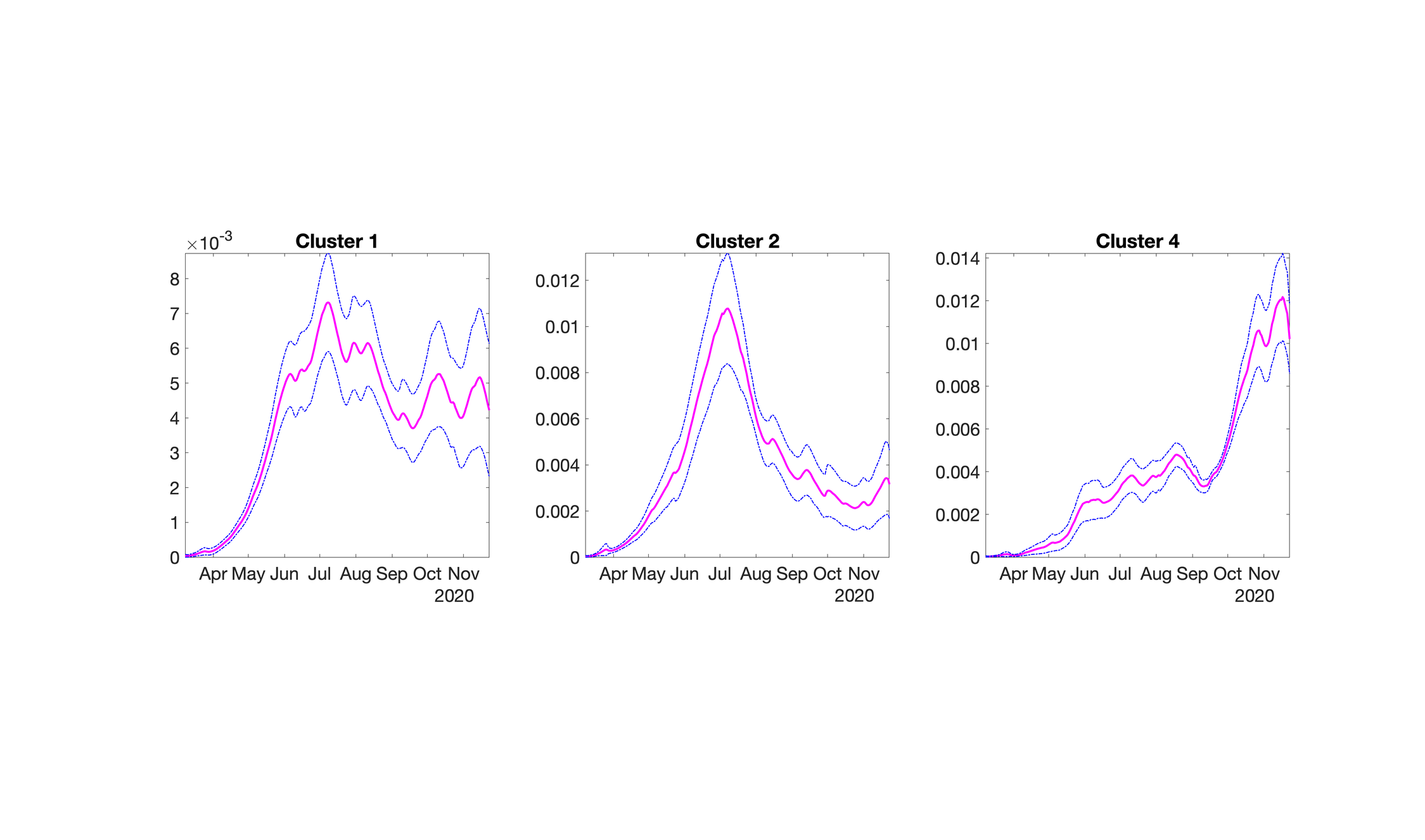

Supplement: S19 Fig — (TIF) [file pone.0254826.s020.tif]

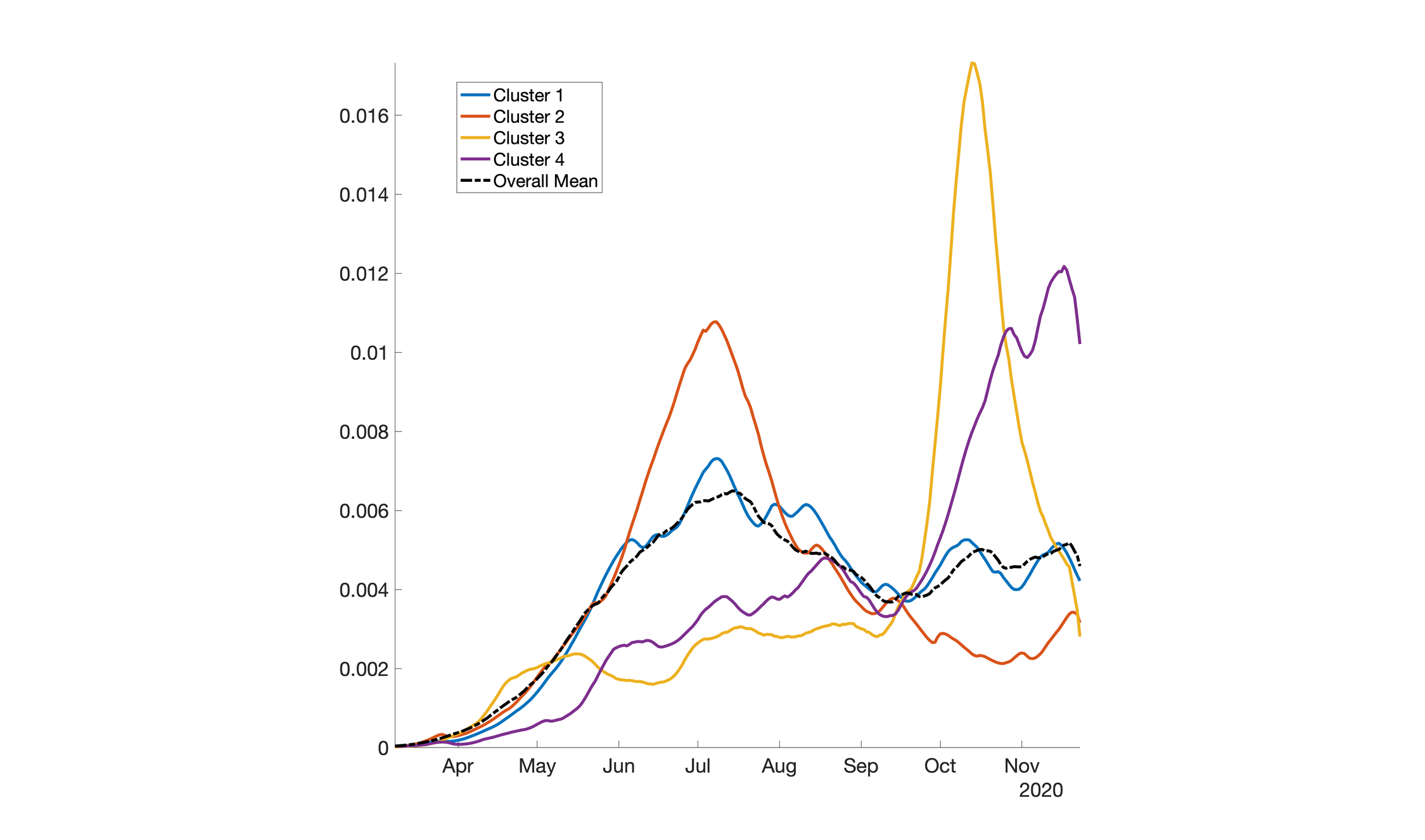

Supplement: S20 Fig — These averages represent the four dominant patterns of incidence rates observed across all states. (TIF) [file pone.0254826.s021.tif]

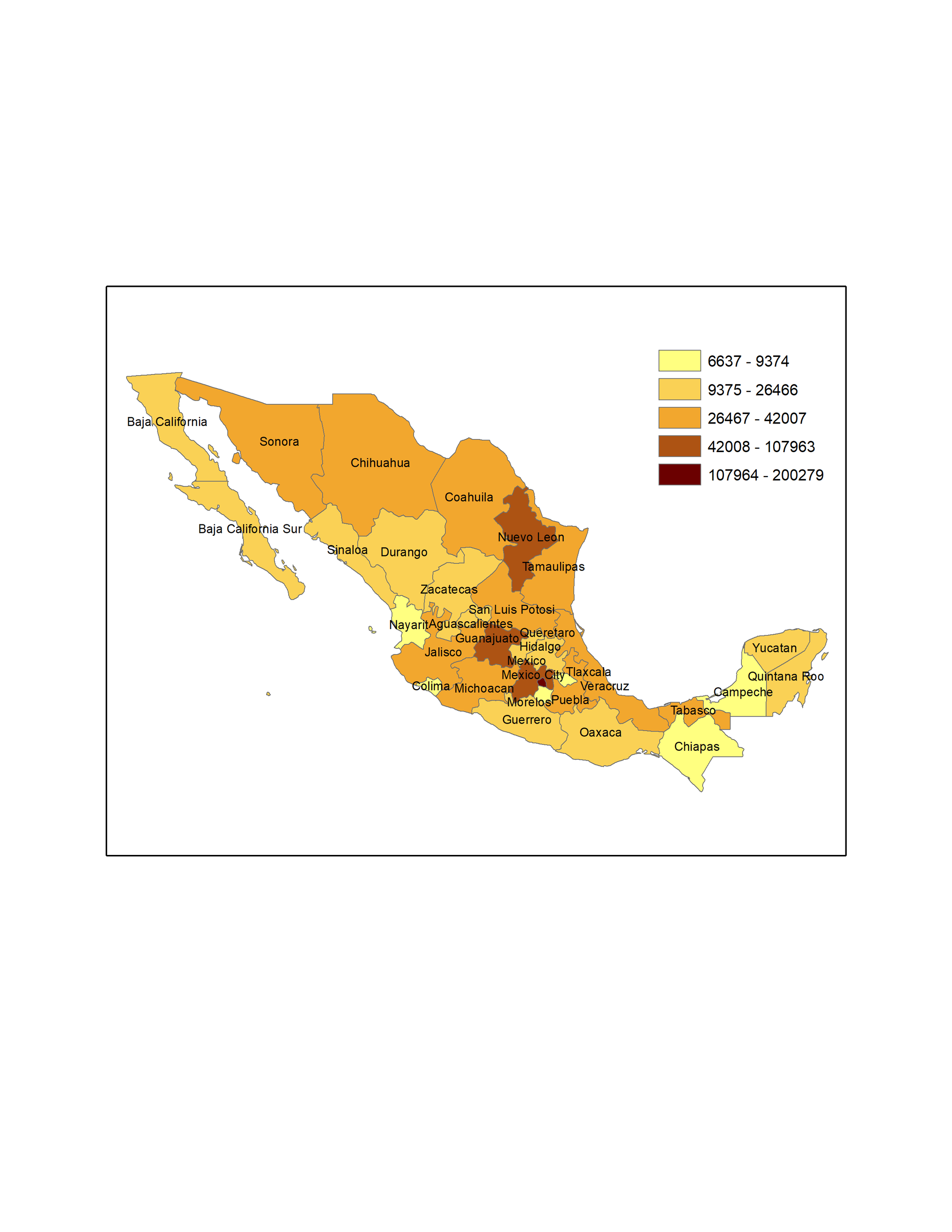

Supplement: S21 Fig — (TIF) [file pone.0254826.s022.tif]

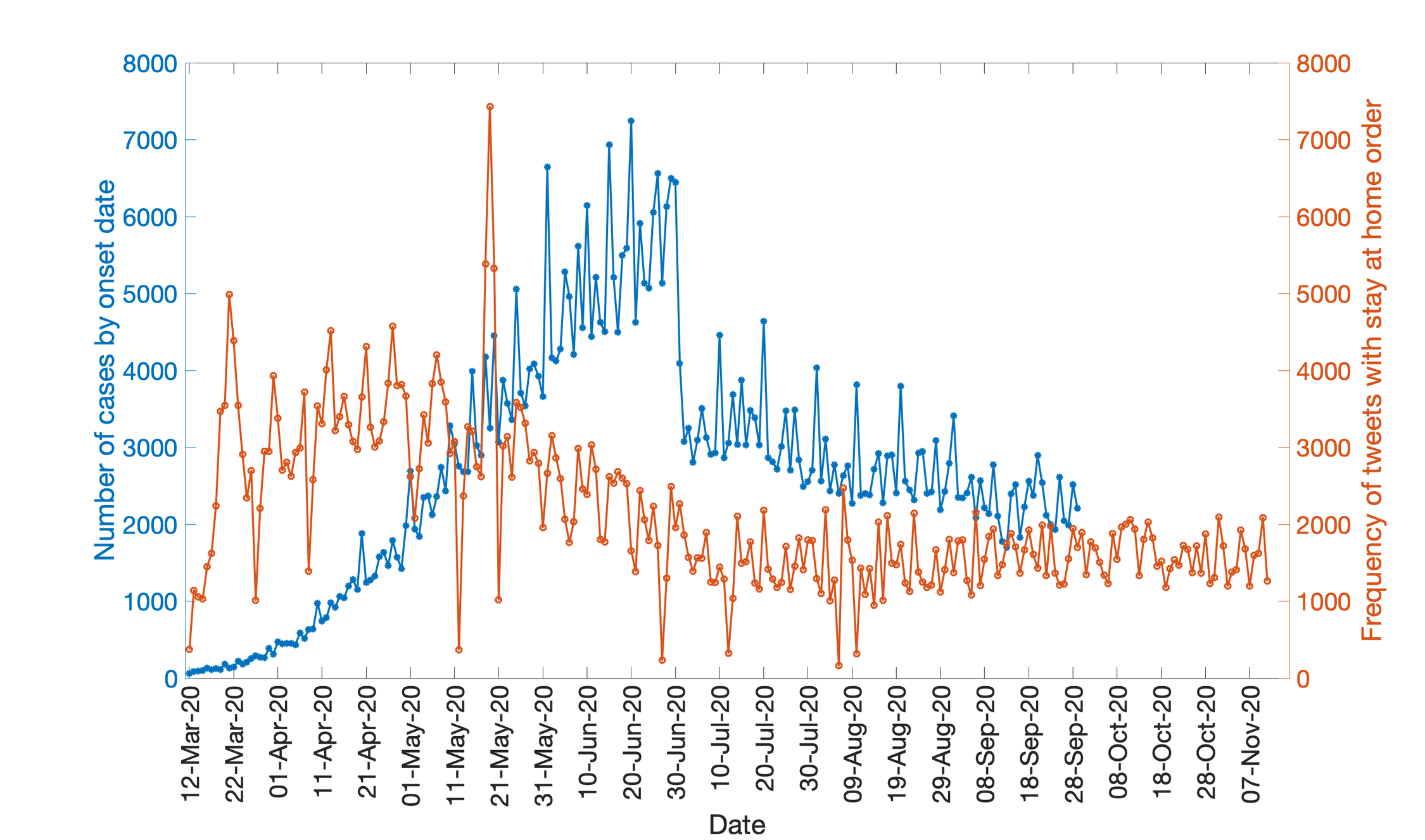

Supplement: S22 Fig — Blue line indicates the number of cases by dates of onset and the orange line indicates the number of tweets referring to the stay-at-home orders. (TIF) [file pone.0254826.s023.tif]
